# Real Space Triplets in Quantum Condensed Matter: Numerical Experiments Using Path Integrals, Closures, and Hard Spheres

**DOI:** 10.3390/e22121338

**Published:** 2020-11-25

**Authors:** Luis M. Sesé

**Affiliations:** Departamento de Ciencias y Técnicas Fisicoquímicas, Facultad de Ciencias, Universidad Nacional de Educación a Distancia (UNED), Avda. Esparta s/n, 28232 Las Rozas, Madrid, Spain; msese@ccia.uned.es

**Keywords:** quantum triplets, path integral Monte Carlo, closures, quantum hard spheres, fluid–solid transition, FCC solid, cI16 solid

## Abstract

Path integral Monte Carlo and closure computations are utilized to study real space triplet correlations in the quantum hard-sphere system. The conditions cover from the normal fluid phase to the solid phases face-centered cubic (FCC) and cI16 (de Broglie wavelengths $0.2\leq \lambda_{B}^{*}<2$, densities $0.1\leq \rho_{N}^{*}\leq 0.925$). The focus is on the equilateral and isosceles features of the path-integral centroid and instantaneous structures. Complementary calculations of the associated pair structures are also carried out to strengthen structural identifications and facilitate closure evaluations. The three closures employed are Kirkwood superposition, Jackson–Feenberg convolution, and their average (AV3). A large quantity of new data are reported, and conclusions are drawn regarding (i) the remarkable performance of AV3 for the centroid and instantaneous correlations, (ii) the correspondences between the fluid and FCC salient features on the coexistence line, and (iii) the most conspicuous differences between FCC and cI16 at the pair and the triplet levels at moderately high densities ($\rho_{N}^{*}=0.9,\;0.925)$. This research is expected to provide low-temperature insights useful for the future related studies of properties of real systems (e.g., helium, alkali metals, and general colloidal systems).

## 1. Introduction

The study of equilibrium triplet structures in 3D *N*-particle systems with quantum behavior remains a pending task in condensed matter research at low temperatures. Apart from a number of early developments focused mainly on the proposal and indirect testing of the so-called *closures* [1,2,3,4,5,6,7,8,9,10,11] or on the use of alternative order parameters [12], just a few computational works based on Feynman’s path integrals (PI) [13] can be found in the recent literature on this field [14,15,16,17,18]. Not much is known about the behavior of quantum triplets, hence the interest in undertaking this task. This is not only a logical step further in current statistical mechanics, allowing one to formulate thermodynamic properties beyond the pairwise approach [19], but also it is central to outstanding condensed matter properties. Among the latter, one can mention the following: phonon–phonon interactions in helium-II [4], the *N*-particle interpretation of fluid entropies [20,21,22], multiple scattering [23], theories of phase transitions [24,25], and glassy dynamics [26,27,28]. Although the whole PI quantum triplet task is computationally daunting at the present time, one can always seek to identify the main triplet features that may serve as a guide for the necessary future work on this topic.

The triplet topic encompasses both real-space {r}–triplets and reciprocal (Fourier)-space {k}–triplets. Nevertheless, there is no direct experimental determination of triplet functions [29,30]. Thus, one must resort to theoretical computations for extracting this sort of information, which makes these computations the only “experimental” method of solving the triplet problem. In the quantum case, a rigorous framework for triplets is given by PI, albeit the computations are truly exacting [14,15,16,17,18]. In this regard, and leaving aside exchange interactions for simplicity, one notes that just the quantum thermal delocalization of the particles is sufficient to bring about a much higher complexity in the quantum study than that present in the classical domain. This helps to understand the key role in this topic that was played by closures, which represent fluid triplet correlation functions $g_{3}\left(r,s,u\right)$ utilizing pair information $g_{2}\left(r\right)$, thus involving affordable computations. Noticeable among the closures for the fluid triplets $g_{3}\left(r,s,u\right)$ are the early Kirkwood superposition KS3 [1,3], and the key Jackson–Feenberg convolution JF3 [4,24], although other forms with even a wider scope are available (e.g., triplet direct correlation functions $c_{3}\left(r,s,u\right))$ [5,24,29]. Despite the fact that closures are approximations to the actual fluid triplet functions, they are still highly valuable in that they may provide insightful physical pictures of the underlying structure of the triplet correlations. Therefore, even nowadays, closures should not be disregarded without giving them the opportunity to prove their worth as interpretative tools in the quantum domain [17,18].

The PI formalism is perfectly suited for performing path integral Monte Carlo (PIMC) and molecular dynamics (PIMD) computer simulations of quantum *N*-particle systems at nonzero temperatures [31,32,33,34,35,36,37,38,39,40,41,42,43,44,45,46,47,48,49,50,51,52,53,54,55]. With due attention to the special characteristics of quantum averages [33,38,44,45,46,47], the latter PI simulations can follow classical-like procedures [56,57,58,59,60]. To illustrate this situation, it is sufficient to recall the most basic PI description in the canonical ensemble (N,V,T) of an actual quantum monatomic system in which exchange interactions can be neglected. Such an actual system is represented by a PI model composed of *N* necklaces with *P* beads apiece, the whole set of N×P beads obeying a classical-like partition function [31,32,33]. It is important to note that *P* is an integer number, P>1, which is to be optimized to obtain statistical convergence for the properties. (The actual quantum system is retrieved in the Trotter’s limit P→∞ [61], while the classical limit is P=1). Special techniques are available to improve the *P* description and reduce computations (e.g., pair actions and higher-order propagators [33,34,35,37,38] combined with algorithms for moving the beads) [33,41,47,62,63]. In this connection, depending on the technique selected, there may or may not exist equivalence between all the beads in the model sample, which is a fact that turns out to be crucial for the study of structures [33,35,38,47]. Thus, one speaks in this context of the structurally significant (equivalent among themselves) beads; their number, say *X*, takes the convenient values *P* or P/2.

The PI applicability covers from quantum diffraction effects (PIMC and PIMD for atomic and molecular fluids and solids [64,65,66,67,68,69]) to bosonic quantum exchange (PIMC) [33,36,41,43]. PIMC and PIMD are said to be “exact” in that they produce results with controllable statistical errors. These results have been proven to be in excellent agreement with experimental data [33,41,42,48]. In addition to this, PIMC approaches to fermionic exchange can also be devised [70], although the “sign problem” precludes the related PIMC applications from being definitive. These facts, together with the PI flexibility, make PIMC and PIMD most powerful tools in quantum condensed matter research.

By focusing attention on quantum monatomic systems at equilibrium, with diffraction effects dominating their behavior, it is worthwhile to specify the three general categories of physically significant structures [31,33,38,39,47] that are revealed by PI: (a) instantaneous; (b) total thermalized-continuous linear response; and (c) centroids (centroid = proper center-of-mass of a PI necklace) [38,47]. For each of these categories, three points must be highlighted [47]. First, a given category is associated with the linear response from the system to a distinct weak external field: the instantaneous case is associated with a δ−localizing field (e.g., as in elastic neutron scattering), this usually being the category linked to “the structure” of a quantum system; the total thermalized-continuous linear response with, for example, a continuous field; and the centroids with specifically a constant-strength field. Second, a given category can be formulated in a two-fold way (real space and Fourier space), which extends over the corresponding *n*-body functions $(g_{n}$ correlation functions and $S^{\left(n\right)}$ static structure factors). Third, a given category is defined by special forms of the averages over the NX bead positions. These averages scale with *X* in different forms: the instantaneous with *X*, the total thermalized-continuous linear response with $X^{n}$, and the centroids with $X^{0}=1$. In stark contrast, the classical system only shows one category of a physically significant structure [56]. Therefore, it is easy to understand the much higher complexity and computational cost of the quantum problem when treated in depth. 

The aim of the present article is to expand the study of the PI triplet features in many-body quantum systems [14,15,16,17,18]. The system selected is that composed of quantum hard spheres (QHS system hereafter). Hard spheres are known to be a very useful reference. They have been used to model classical and quantum systems, ranging from helium [33,36,66,67,71,72,73] to complex colloids [74,75], and under very different conditions (i.e., fluid, boson superfluid, superlattices, solids, and suspensions). This work concentrates on the real space instantaneous and centroid triplets, leaving aside the total thermalized-continuous linear response case to keep the related computations affordable [17]. As stressed earlier, in understanding triplets through closures, a thorough consideration of the pair structures is needed. This benefits the triplet-closure computations and the analysis of the correspondences between the salient pair and triplet features (in different phases or within the same phase). Therefore, the necessary attention is also paid to the pair prerequisite.

The exact computational method chosen is PIMC, which avoids the PIMD difficulties linked to the discontinuity of the hard-sphere potential, and it is complemented with the closures: KS3, JF3, and their average AV3 = (KS3 + JF3)/2. A wide range of QHS fluid and solid conditions, within the purely diffraction regime, is studied: reduced de Broglie wavelengths $0.2\leq \lambda_{B}^{*}<2$ and reduced number densities $0.1\leq \rho_{N}^{*}\leq 0.925$, where $\lambda_{B}^{*}=h/{\left(2\pi Mk_{B}T\sigma^{2}\right)}^{1/2}$, $\rho_{N}^{*}=N\sigma^{3}/V$, σ and M being the hard-sphere diameter and mass, respectively. The specific {r}-space targets pursued are the following:

(i) Analyzing in detail the potential usefulness of AV3 for quantum fluid triplet studies. This is prompted by the encouraging results obtained in [17] for liquid para-hydrogen and liquid neon.
(ii) Gaining triplet structural insights from the comparison, in the short–medium range of distances, between the coexisting fluid and FCC (face-centered cubic) solid [66,67].
(iii) Comparing the salient triplet features of the cubic solids FCC and cI16 at moderately high densities, ($\rho_{N}^{*}$ = 0.925, $\lambda_{B}^{*}$ = 0.2) and ($\rho_{N}^{*}$ = 0.9, $\lambda_{B}^{*}$ = 0.8). The so-called cI16 lattice is a distorted superstructure of the body-centered cubic (BCC) lattice [76,77]. (Hard-sphere BCC lattices are known to be mechanically unstable in both classical and quantum applications [78,79]). One also notes that cI16 phases have been reported for Li and Na at very high pressures [76,77], hence the interest of this point.
(iv) In connection with (iii), there is the question of establishing a clear identification of the QHS bcc-qIII phases, observed in [67,78], as genuine cI16 phases. (The bcc-qIII phases arise from the PIMC evolution of initially perfect BCC lattices). The cI16 lattice has been identified recently for classical hard spheres in the insightful simulation work reported in [80], and the patterns of the related results suggest that bcc-qIII is in fact cI16. Proof of this is given in this article, which adds more value to the QHS system for modeling quantum solid–solid changes of phase [67] and enhances the meaning of the related triplet calculations.

It is hoped that the reported “experimental” results will form a useful basis for comparison when extensive studies of triplets in real quantum systems are undertaken. The outline of this article is as follows. Section 2 contains the theoretical background. Section 3 describes the relevant computational details. Section 4 gives the results and their discussion, and Section 5 collates the main conclusions of this work.

## 2. Theory

### 2.1. Path Integral Monte Carlo (PIMC)

The canonical partition function Z(N,V,T) of a quantum monatomic system (number density $\rho_{N}=N/V)$, under conditions where exchange interactions can be neglected, can be approximated by the accurate PI formula (Tr= trace) [31,32,33]

(1) $$Z=Tr\left\{\exp\left(-\beta H_{N}\right)\right\}≅\frac{1}{N!}{\left(\frac{MP}{2\pi \beta \hbar^{2}}\right)}^{3NP/2}\int \;\prod_{j=1}^{N}\prod_{t=1}^{P}dr_{j}^{t}\times exp\left[-\beta W_{NP}\right],$$

where $H_{N}$ is the Hamiltonian, assumed normally to be composed of one- and two-particle terms, M is the particle mass, $\beta =1/k_{B}T$, P is the discretization in beads of the necklace representing and actual particle j, $r_{j}^{t}$ denotes the real space coordinates of bead t (t=1,2,…,P) belonging to necklace j, and $W_{NP}$ is the “effective potential” ruling the whole set of N×P beads (hereafter all of them equivalent: X=P). In what follows, the optimal P will be assumed. In addition, it is worthwhile to note that (a) consecutive beads, t and t+1, in a necklace are separated by βℏ/P in Euclidean imaginary time βℏ; (b) then, a given bead labeled t is associated with the imaginary time βℏt/P; and (c) the cyclic property t+1=P+1≡1 is satisfied.

In the case of the QHS system, an appropriate choice for $W_{NP}$ is based on Cao–Berne’s CBHSP pair action [35], and it can be written as [14,78]

(2) $$W_{NP}^{CBHSP}=W_{1}^{F}+W_{2}^{CB}+W_{2}^{HS},$$

(3a) $$W_{1}^{F}=\frac{MP}{2\beta^{2}\hbar^{2}}\sum_{j=1}^{N}\sum_{t=1}^{P*}{\left(r_{j}^{t}-r_{j}^{t+1}\right)}^{2},$$

(3b) $$W_{2}^{CB}=-\beta^{-1}ln\prod_{j<m}\prod_{t=1}^{P*}\left\{1-\frac{\sigma \left(r_{jm}^{t}+r_{jm}^{t+1}-\sigma \right)}{r_{jm}^{t}r_{jm}^{t+1}}\times exp\left[-\frac{MP\left(r_{jm}^{t}-\sigma \right)\left(r_{jm}^{t+1}-\sigma \right)}{2\beta \hbar^{2}}\left(1+\cos\left(r_{jm}^{t},r_{jm}^{t+1}\right)\right)\right]\right\},$$

(3c) $$W_{2}^{HS}=P^{-1}\sum_{j<m}\sum_{t=1}^{P}\omega_{HS}(r_{jm}^{t})=\left\{\begin{array}{c} \infty \;\;if\;\;\;r_{jm}^{t}=\left|r_{j}^{t}-r_{m}^{t}\right|<\sigma \;\\ 0\;\;\;if\;\;\;\;\;\;\;\;\;\;\;\;\;\;\;\;\;\;\;\;\;\;\;\;\;\;r_{jm}^{t}>\sigma \end{array}\right\}.\;$$

In Equation (3a), one recognizes the superposition of the free-particle behaviors [13]. Equation (3b) shows Cao–Berne’s correction, where the adjacent-bead effects are to be noted. Equation (3c) is the expression of the singular hard-sphere potential extended over all the pairs of necklaces, which interact in an equal “t–time” bead-to-bead fashion (ET). The symbols P∗ in the sum and product above mark the t−cyclic property already mentioned. For the specific thermodynamic property formulas that can be derived from CBHSP, the reader is referred to [67,78]. At this point, it is important to give the definition of the CBHSP centroid of a given necklace j

(4) $$R_{CM,j}=P^{-1}\sum_{t=1}^{P}r_{j}^{t}.$$

This quantity plays an important role in PI theoretical developments [13,47], in particular in (a) the appealing centroid approaches to quantum dynamics [81,82,83,84,85]; (b) the exact formulation of the equation of state of quantum fluids [39,86]; and (c) the characterization of quantum solid phases [67].

A key feature of the QHS system is that its state points can be uniquely characterized by giving two parameters, namely the reduced number density $\rho_{N}^{*}$ and the reduced de Broglie thermal wavelength $\lambda_{B}^{*}$. This fact was early realized at the level of semiclassical treatments based on ℏ–expansions (see, for instance, [87,88,89,90]). Within PI, the same fact is just a property contained in the mathematical structure of the QHS partition function, regardless of the particular proper form that $W_{NP}$ may take (see [47] for a discussion of QHS propagators). Accordingly, the QHS system properties can be expressed in reduced units, thereby being independent of the actual parameters M, σ, T, and $\rho_{N}$ employed [66,67]. Therefore, for example, internal energies E can be given as $E^{*}=E/RT$, and pressures p can be given as $p^{*}=pM\sigma^{5}/\hbar^{2}$. For the pressure, an indication to guide the interested reader will suffice: when using different sets of parameters to define the state points 1 and 2, if ${\left(\rho_{N}^{*},\lambda_{B}^{*}\right)}_{1}={\left(\rho_{N}^{*},\lambda_{B}^{*}\right)}_{2}$, then necessarily ${\left(PV/RT\right)}_{1}={\left(PV/RT\right)}_{2}$, and also $p_{1}^{*}=p_{2}^{*}$.

The same general type of argument applies to the real space structures $g_{n}\left(q_{1},q_{2},\ldots ,q_{n}\right)$, for which, when reporting QHS system results, it is most useful to do it using interparticle distances in reduced form: $r_{12}^{*}=\left|q_{1}-q_{2}\right|/\sigma$. In order not to burden the notation, the formulation of the structural concepts below will utilize the distances and related quantities in their non-reduced forms, as in Equations (1) to (4).

Another technical point seems worth placing here. It is related to the three- (and higher-order) particle contributions to the quantum Hamiltonian $H_{N}$ of the system, which may yield more complete and elaborate forms for the propagators and $W_{NP}$. While this is a question that can be tackled in various ways when continuous interparticle potentials are involved [91,92,93], to this author’s knowledge, no QHS system PI actions beyond the pair level are available, and such an extension remains intractable for now. Nevertheless, because of the strong similarity between helium atoms and quantum hard spheres, the related effects on the QHS system can be expected to become significant at very high solid-phase densities (and sufficiently low temperatures) [33]. Furthermore, owing to the QHS infinite repulsion at the hard core, Equation (3c), the wave functions of the QHS system must vanish for interparticle distances r≤σ (i.e., there can be no tunneling); hence, quantum hard spheres repel one another before “classical contact” can occur [89,90]. (Within PI, this means that the related forbidden region brought about by Equation (3c) arises only for the “equal-time” bead $g_{n}$ correlations). Therefore, given the lack of any attraction, the “preemptive” QHS repulsions can be expected to cause a strong impediment to the coming together of triplets of quantum hard spheres. Using the quantum diffraction parameter $\gamma =\rho_{N}^{*}\lambda_{B}^{*3}$, the latter triplet effects should not play any significant role unless γ becomes truly high. The largest value of γ in this work is ≅2.8, which is compatible with the QHS pair modeling of normal fluid and solid helium-4 [66]. Therefore, the pair-level CBHSP approach can be deemed adequate to compute structures under the fluid and solid conditions investigated in this work.

### 2.2. PI Triplet Structures

Within PI-CBHSP, the centroid (CM3) and the instantaneous (ET3) three-point number densities can be cast as the ensemble averages [17]

(5) $$\rho_{CM3}^{\left(3\right)}\left(q_{1},q_{2},q_{3}\right)=\langle \sum_{j\neq l\neq m\neq j}\delta \left(R_{CM,j}-q_{1}\right)\delta \left(R_{CM,l}-q_{2}\right)\delta \left(R_{CM,m}-q_{3}\right)\rangle ,$$

(6) $$\rho_{ET3}^{\left(3\right)}\left(q_{1},q_{2},q_{3}\right)=P^{-1}\langle \sum_{t=1}^{P}\sum_{j\neq l\neq m\neq j}\delta \left(r_{j}^{t}-q_{1}\right)\delta \left(r_{l}^{t}-q_{2}\right)\delta \left(r_{m}^{t}-q_{3}\right)\rangle ,$$

where one notices that (i) the multi-index summations run over the whole set of permutations of N particles taken three at a time; (ii) the instantaneous case contains a further P average involving “equal-time” beads in different necklaces; and (iii) these definitions are completely general, since they depend on the position vectors of the representative set of three particles and can be applied to the statistical description of monatomic systems, which are either fluid or solid. Due to the high computational cost, no attempt is made in this work at calculating total thermalized-continuous linear response triplets [14,17].

For homogeneous and isotropic fluids, one finds simpler formulas [17]

(7) $$\rho_{CM3}^{\left(3\right)}\left(q_{1},q_{2},q_{3}\right)=\rho_{N}^{3}g_{CM3}\left(q_{1}-q_{3},q_{2}-q_{3}\right)=\rho_{N}^{3}g_{CM3}\left(r_{12},s_{13},u_{23}\right),$$

(8) $$\rho_{ET3}^{\left(3\right)}\left(q_{1},q_{2},q_{3}\right)=\rho_{N}^{3}g_{ET3}\left(q_{1}-q_{3},q_{2}-q_{3}\right)=\rho_{N}^{3}g_{ET3}\left(r_{12},s_{13},u_{23}\right),$$

where the triplet correlation functions $g_{CM3}$ and $g_{ET3}$ depend only on the three generic interparticle distances: $r_{12}=\left|q_{1}-q_{2}\right|$, $s_{13}=\left|q_{1}-q_{3}\right|$, and $u_{23}=\left|q_{2}-q_{3}\right|$. This exact reduction from nine to three independent variables makes the intricate triplet problem more accessible for the study of monatomic fluid state points [14,15,16,17].

Rigorously speaking, the related exact framework for a monatomic solid is contained in Equations (5) and (6). Nevertheless, affordable approximations to this even more costly problem can be obtained by applying Equations (7) and (8). Actually, such an approach is consistent with the same idea, already exploited successfully, at the pair level in the study of regular solid phases, since the $g_{CM2}\left(r\right)$ and $g_{ET2}\left(r\right)$ pair radial functions retain many significant traits of the underlying solid structure [66,67,80]. Furthermore, as a first step, the use of Equations (7) and (8) facilitates the comparison of the global salient triplet features appearing in different solid phases.

The functions defined in Equations (7) and (8) must satisfy several properties [4,6,7,57,58]. The most relevant to this work are:

(1) Symmetry
  (9a) $$g_{3}\left(q_{1},q_{2},q_{3}\right)=g_{3}\left(q_{2},q_{3},q_{1}\right)=\ldots ;\;\mathrm{ET}3\;\mathrm{and}\;\mathrm{CM}3.$$
(2) QHS instantaneous behavior at close distances
  (9b) $$\underset{\left|q_{j}-q_{m}\right|\to \sigma +}{\lim}g_{ET3}\left(r,s,u\right)=0.$$
(3) Asymptotic behavior in fluids
  (9c) $$\underset{r\to \infty }{\lim}g_{3}\left(r,r,r\right)=1;\;\mathrm{ET}3\;\mathrm{and}\;\mathrm{CM}3,$$
  (9d) $$\underset{s\to \infty }{\lim}g_{3}\left(r,s,s\right)=g_{2}\left(r\right);\;\mathrm{ET}3/\mathrm{ET}2\;\mathrm{and}\;\mathrm{CM}3/\mathrm{CM}2.$$

Equation (9a) follows from Equations (7) and (8). Equation (9b) for the instantaneous case arises from the singular character of the hard-sphere potential Equation (3c). For centroids, a behavior similar to Equation (9b) is expected to occur, albeit the limiting distance may be different from σ. Finally, Equations (9c) and (9d) follow from the weakening of particle correlations in fluids when considering increasing distances, and both are very useful to check the inner consistency of the related calculations.

### 2.3. Additional Pair Structural Quantities

To supplement the PIMC triplet calculations in the canonical ensemble, the following quantities can also be computed:

(a) The pair radial functions for the centroid (CM2) and the instantaneous (ET2) correlations, in both the fluid and the solid phases [47]. Their PI ensemble averages can be cast as
  (10) $$\rho_{N}^{2}g_{CM2}\left(r_{12}\right)=\langle \sum_{j\neq m}\delta \left(R_{CM,j}-q_{1}\right)\delta \left(R_{CM,m}-q_{2}\right)\rangle ,$$
  (11) $$\rho_{N}^{2}g_{ET2}\left(r_{12}\right)=P^{-1}\langle \sum_{t=1}^{P}\sum_{j\neq m}\delta \left(r_{j}^{t}-q_{1}\right)\delta \left(r_{m}^{t}-q_{2}\right)\rangle ,$$
  where $r_{12}=\left|q_{1}-q_{2}\right|$.
(b) The pair static structure factors $S_{CM}^{\left(2\right)}\left(k\right)$ and $S_{ET}^{\left(2\right)}\left(k\right)$ associated with the foregoing pair radial structures in the fluid phase [47]
  (12) $$S_{CM}^{\left(2\right)}\left(k\right)=1+\rho_{N}\int^{\;}dr_{12}\exp\left(ik\cdot r_{12}\right)h_{CM2}(r_{12})={\left(1-\rho_{N}c_{CM}^{\left(2\right)}\left(k\right)\right)}^{-1},$$
  (13) $$S_{ET}^{\left(2\right)}\left(k\right)=1+\rho_{N}\int^{\;}dr_{12}\exp\left(ik\cdot r_{12}\right)h_{ET2}(r_{12})≅{\left(1-\rho_{N}c_{ET}^{\left(2\right)}\left(k\right)\right)}^{-1},$$
  where $h_{2}=g_{2}-1$ stands for the corresponding pair *total* correlation function, and $c^{\left(2\right)}\left(k\right)$ stands for the corresponding pair *direct* correlation function in Fourier space (k=|k|). These structure factors can be fixed with great accuracy, at a very low cost and for every k≥0 wave number [48], via the Ornstein–Zernike framework [94,95,96] developed by this author [47,86,97,98]. Apart from their intrinsic usefulness, they are decisive in achieving a number of significant improvements in the study of fluids with quantum behavior [39,47,48,49]. In particular, $S_{CM}^{\left(2\right)}\left(k\right)$ and $S_{ET}^{\left(2\right)}\left(k\right)$ can be utilized for (i) extending the ranges of the simulated $g_{CM2}\left(r_{12}\right)$ and $g_{ET2}\left(r_{12}\right)$ [47], which serves to perform triplet closure computations; and (ii) gaining insight into their associated triplet structure factors $S_{CM}^{\left(3\right)}\left(k_{1},k_{2}\right)$ and $S_{ET}^{\left(3\right)}\left(k_{1},k_{2}\right)$ [17,18].
(c) In simulation work using cubic boxes, the PI sample size is composed of $N_{S}$ necklaces, each with P beads, enclosed in a volume $V_{S}=L^{3}$. To characterize solid phases, one can employ the normalized-to-unity solid-phase configurational structure factors at the centroid and instantaneous pair levels [67,99,100]. They can be written as
  (14) $$S_{CM2}^{\left(C\right)}\left(k\right)=N_{S}^{-2}{\left|\sum_{j=1}^{N_{S}}\exp\left(ik\cdot R_{CM,j}\right)\right|}^{2},$$
  (15) $$S_{ET2}^{\left(C\right)}\left(k\right)={\left(N_{S}^{2}P\right)}^{-1}\sum_{t=1}^{P}{\left|\sum_{j=1}^{N_{S}}\exp\left(ik\cdot r_{j}^{t}\right)\right|}^{2},$$
  and are taken at their maximal values arising from the simulation runs [67,78]. In these simulation conditions, the wave vectors k to be analyzed must be commensurate with the box, which means $k=2\pi L^{-1}\left(k_{x},k_{y},k_{z}\right)$, where the components $\left(k_{x},k_{y},k_{z}\right)$ take integer values [56]. In connection with this, notice that cubic-based *perfect* lattices can be associated with sets of three commensurate wave vectors, ${\left\{k_{w}\right\}}_{n}={\left\{k_{1},k_{2},k_{3}\right\}}_{n}$, which are maximal in that:

(i) For the perfect FCC and BCC lattices, one can single out sets ${\left\{k_{w}\right\}}_{n}$ such that they reach the maximum value, $S_{2}^{\left(C\right)}\left(k_{w}\right)=S_{2}^{\left(C\right)}\left(k_{max}\right)=1$, (w=1,2,3). For a perfect cI16 lattice, which is not so highly regular, one obtains $S_{2}^{\left(C\right)}\left(k_{w}\right)=S_{2}^{\left(C\right)}\left(k_{max}\right)<1$, (w=1,2,3), as will be shown later on.
(ii) The following result holds
  (16) $$\left|k_{1}\cdot \left(k_{2}\times k_{3}\right)\right|={\left(2\pi \right)}^{3}N_{S}/V_{S}.$$

Therefore, comparison of the above standard perfect-lattice results with those arising from the simulated cubic solid phase allows one to identify its type and relative order. Obviously, the values of the simulated configurational structure factors are lower than the perfect reference values; they appear associated with each of the three maximal wave vectors and are close to one another, but, as a rule, they are not equal: one of them can be singled out as the maximum, whereas the other two remain slightly below [67,78]. As a guide for quantum work [99], the following *centroid* estimates are worth quoting: $0.4≲S_{CM2}^{\left(C\right)}\left(k_{max}\right)$ for partially crystalline solids, while typically $S_{CM2}^{\left(C\right)}\left(k_{max}\right)<0.2$ for fluid phases. (Amorphous phase maximal values for $S_{CM2}^{\left(C\right)}\left(k_{max}\right)$ should be between the two foregoing limits). It is important to stress that although somewhat expensive to calculate, the quantities $S_{CM2}^{\left(C\right)}\left(k_{max}\right)$ and $S_{ET2}^{\left(C\right)}\left(k_{max}\right)$ are global for the simulation sample. Therefore, in this context, these quantities seem more complete than local-order parameters (e.g., the rotationally invariant $Q_{l})$ [67,80,101].

Before going any further, it is convenient to consider the general issue of the simulation sample size $N_{S}$ for the solid phases, thus allowing one to introduce cI16 basic details. The conditions for FCC and BCC are well-known, and for $N_{S}>100$, one finds: (i) $N_{S}\left(\mathrm{FCC}\right)=4n^{3}$, with n=3,4,5,…; and (ii) $N_{S}\left(\mathrm{BCC}\right)=2n^{3}$, with n=4,5,6,…. However, the case of cI16 is not so standard. cI16 is a distortion of BCC and is characterized by the so-called *fractional displacement parameter*, which is usually denoted by x [76,77], so as to have the particles occupying the 16c Wyckoff site (x,x,x) of the space group $I\bar{4}3d$. This means that its body-centered unit cell does contain 16 particles. Consequently, there are some extra restrictions that may make the $N_{S}\left(\mathrm{cI}16\right)$ values different from those of BCC. Thus, again for $N_{S}>100,$ one finds (iii) $N_{S}\left(\mathrm{cI}16\right)=16n^{3}$, with n=2,3,4,…. The reader is referred to [76,77,80] for specific details.

### 2.4. Closures for Fluid Triplets

The two basic closures analyzed in this work are Kirkwood superposition KS3 and Jackson–Feenberg convolution JF3. Both can be applied to the fluid centroid (CM3) and instantaneous (ET3) triplet correlations. Their expressions can be written as follows [1,4]:

(17) $$g_{KS3}\left(r_{12},s_{13},u_{23}\right)=g_{2}\left(r_{12}\right)g_{2}\left(s_{13}\right)g_{2}\left(u_{23}\right),$$

(18) $$g_{JF3}\left(r_{12},s_{13},u_{23}\right)=g_{KS3}\left(r_{12},s_{13},u_{23}\right)-h_{2}\left(r_{12}\right)h_{2}\left(s_{13}\right)h_{2}\left(u_{23}\right)+\;\rho_{N}\int^{\;}dq_{4}h_{2}\left(v_{14}\right)h_{2}\left(v_{24}\right)h_{2}\left(v_{34}\right),$$

where $v_{j4}=\left|q_{j}-q_{4}\right|$, $h_{2}=g_{2}-1$, and $g_{2}=g_{CM2}$ or $g_{ET2}$. Although explicitly stated in Equation (18), it is important to remark that JF3 lacks the triplet-product term $h_{2}\left(r_{12}\right)h_{2}\left(s_{13}\right)h_{2}\left(u_{23}\right)$, which should appear in an $h_{2}-$expansion. This absence has deep consequences as will be shown in this article. An easy and direct way to recover such contribution (half of it), while at the same time keeping the convolution integral (half of it) contained in Equation (18), is via the average closure AV3 that reads as

(19) $$g_{AV3}\left(r_{12},s_{13},u_{23}\right)=\frac{1}{2}\left(g_{KS3}\left(r_{12},s_{13},u_{23}\right)+g_{JF3}\left(r_{12},s_{13},u_{23}\right)\right).$$

As regards the properties of these closures, suffice it to say that (i) KS3, JF3, and AV3, satisfy Equations (9a), (9c), and (9d); and (ii) only KS3, as induced by its construction, satisfies Equation (9b), which is a special case of the general triplet behavior $g_{3}\to 0$ when two particles approach closely each other [14,15,16,17].

## 3. Computational Details

The main target of this work is the determination of QHS equilateral and isosceles triplet correlations (centroid and instantaneous), namely the types of functions $g_{3}\left(r,r,r\right)$ (or $g_{3}^{EQ}$ for brevity when necessary) and $g_{3}\left(r,s,s\right)$. For the sake of interpretation, they are complemented with the additional structural properties discussed in Section 2.3. The state points studied are shown in Table 1. They span a wide range of conditions, from the normal fluid phase to the distinct solid phases FCC and cI16. Special attention is paid to the two sides of the fluid–FCC coexistence line, as determined in [67] $\left(\lambda_{B}^{*}\leq 0.8\right)$ and [66] $\left(\lambda_{B}^{*}>0.8\right)$. Moreover, the study is extended to (i) fluid state points under very strong diffraction effects $\left(\lambda_{B}^{*}\approx 2\right)$, with a view to establishing a meaningful correlation of triplet structures when going toward the change of phase by increasing $\rho_{N}^{*}$ at constant temperature, and (ii) the lattices FCC and cI16 at $\left(\rho_{N}^{*}=0.925,\;\lambda_{B}^{*}=0.2\right)$ and $\left(\rho_{N}^{*}=0.9,\;\lambda_{B}^{*}=0.8\right)$, which are conditions that are significantly far from the very high-density regions.

### 3.1. PIMC Calculations

The PIMC simulation procedures utilized can be found elsewhere [14,15,16,17,67,78], although for completeness, the basic lines follow below.

The necklace normal mode algorithm [62,63] is used to generate the collective P movements of a given necklace, with a Metropolis acceptance ratio of 50%. (As in previous works, the actual hard-sphere parameters are M=28.0134 amu and σ=3.5 Å; $1\;Å=10^{-10}\;m)$. The necklace sample sizes $N_{S}$ are 864 for the fluid and the FCC solid phases, and 1024 for the cI16 solid phases. The quantum P convergence for the results is studied as shown below (12≤P≤40). One kpass is defined as a set of $10^{3}N_{S}\times P$ attempted bead moves, and one Mpass is then $10^{3}$ kpasses. After equilibration, most of the simulation runs are arranged into 40 blocks for the $g_{2}$ calculations and 30 blocks for the $g_{3}$ calculations. The respective block sizes are (i) 92.6 kpasses for the fluid simulations; (ii) 92.6 kpasses for the FCC simulations; and (iii) 78.125 kpasses for the cI16 simulations. Therefore, the run lengths associated with the $g_{2}$ and $g_{3}$ calculations are in between 2.34 Mpasses and 3.7 Mpasses. (The extra simulations using P=36 and 40 have lengths of about 1 Mpass). Block subaverages for $g_{2}$ and $g_{3}$ are obtained by gathering statistics every 5000 $\left(g_{2}\right)$/7000–8000 $\left(g_{3}\right)$ configurations generated. The configurational structure factors given by Equations (14) and (15) are analyzed four times per block, at equally spaced intervals, by recording the ten largest values for the final assessment. To do so, triplets of integers $\left(k_{x},k_{y},k_{z}\right)$ are monitored in the mesh $25\leq k_{x}^{2}+k_{y}^{2}+k_{z}^{2}\leq 200$, with the components in $-10\leq k_{\nu }\leq 10$ (symmetry properties allow one to reduce the calculations). Given that the information provided by the correlation functions, complemented with that arising from the structure factors, is more than sufficient to characterize the current solid structural results, the $Q_{l}$ order parameters [101] are not evaluated, thereby alleviating the considerable computational effort involved in this work.

The pair and triplet sructures $g_{2}$ and $g_{3}$ are fixed in the established ways using histograms. The case of $g_{2}$ is straightforward and well-known [56], and the simulations are utilized as the reduced width of the bins ${\bar{\Delta }}^{*}=1/35$ (or σ/35=0.1 Å). However, the case of $g_{3}$ includes a good number of subtleties [57,58]. The detailed description of the related procedure can be found in [14]. For the current purposes, suffice it to say that for a triplet of distances (R,S,U), the basic $g_{3}$ expression is given by

(20) $$g_{3}\left(R,S,U\right)=\frac{\left(\Delta n_{T}\right)}{N\rho_{N}^{2}{\left(\Delta V\right)}_{RSU}};\;\mathrm{ET}3\;\mathrm{and}\;\mathrm{CM}3$$

where $\left(\Delta n_{T}\right)$ is the number of times mutual distance triplets lie within the ranges $R-\bar{\Delta }<r_{12}\leq R$, $S-\bar{\Delta }<s_{13}\leq S$, and $U-\bar{\Delta }<u_{23}\leq U$, and ${\left(\Delta V\right)}_{RSU}$ stands for the appropriate volume element [58]. Once again, in these calculations, ${\bar{\Delta }}^{*}=1/35$. The histogramming of triplets extends up to distances $r_{12}$, $s_{13}$, and $u_{23}$, which are <L/4. Statistical errors (one-standard deviation) for the average structures computed with PIMC are fixed with the corresponding block subaverages. For example, for the first peaks heights of $g_{2}$ and $g_{3}$, the errors remain below 1% for most of the present calculations. In this connection, Table 2 gives some representative $g_{3}$ results (mean first peaks (FP)) in the close vicinities of the absolute maxima of the structure indicated, together with the associated errors. (More on this in the Supplementary Materials). Note that the P convergence is influenced by both $\lambda_{B}^{*}$ and $\rho_{N}^{*}$. For the fluid and FCC state points on the coexistence line, under the most extreme quantum conditions studied herein $(\lambda_{B}^{*}=1.9832,\;\gamma ≅2.7-2.8$), P=30 is sufficient to produce practical convergences in the centroid and in the instantaneous functions. For the solid state points at densities $\rho_{N}^{*}=0.9,\;0.925$, it is worthwhile to note that there is a slowing down of this convergence with decreasing temperatures $\left(\lambda_{B}^{*}=0.2\to 0.8\right)$, which becomes more noticeable (a) for the triplet centroid quantities and (b) for the cI16 lattice, its openness playing a significant role in the fixing of the final particle distributions.

### 3.2. Closure Calculations

The current calculations at the actual fluid state points on the coexistence line use the new PIMC information obtained with sample sizes larger than those employed in [49]. (The new and the former results are in excellent agreement). The JF3 convolution integrals involve the $h_{2}\;$extension to longer distances fixed with the fluid $S_{CM}^{\left(2\right)}\left(k\right)$ and $S_{ET}^{\left(2\right)}\left(k\right)$. The convolutions can be obtained by employing a well-known expansion in Legendre polynomials $P_{n}$ [10,24]

(21a) $$\int^{\;}dq_{4}h_{2}\left(\upsilon_{14}\right)h_{2}\left(\upsilon_{24}\right)h_{2}\left(\upsilon_{34}\right)≅\sum_{n=0}^{n_{max}}\pi \left(2n+1\right)P_{n}\left(cos\phi \right)I_{n}\left(h_{2},P_{n}\right),$$

(21b) $$I_{n}\left(h_{2},P_{n}\right)=\int_{0}^{y_{max}}dy\;y^{2}h_{2}\left(y\right)f_{n}\left(y,s_{13}\right)f_{n}\left(y,u_{23}\right),$$

(21c) $$f_{n}\left(y,z\right)=\int_{-1}^{+1}dx\;P_{n}\left(x\right)h_{2}\left(\sqrt{y^{2}+z^{2}-2xyz}\right),$$

where ϕ is the angle between $s_{13}$ and $u_{23}$. The final JF3 results reported in Section 4 employ (a) $n_{max}=30$ for the Legendre expansion; (b) $y_{max}=20\sigma =70\;Å$ (i.e., $y_{max}^{*}=20)$ as the maximum distance for $h_{2}$ data; and (c) trapezoidal quadratures with discretizations consisting of 2000 points for the y integrations and 1000 points for the x integrations. The latter parameters are sufficient to yield JF3 and AV3 results that can be compared with PIMC in a meaningful way. To grasp this point, some results at the highest-density fluid state point $\left(\rho_{N}^{*}=0.789,\;\lambda_{B}^{*}=0.2\right)$ will suffice. The JF3 centroid (CM3) and instantaneous (ET3) results in the close vicinities $r_{FP}^{*}$ (first peaks FP) of their respective equilateral (EQ) absolute maxima, $(r_{FP}=3.85\;Å$, or $r_{FP}^{*}=1.1)$, behave as follows. (i) $n_{max}=10$, $y_{max}=50\;Å$ ($y_{max}^{*}\approx 14.3)$, using 1000-point y integration, plus 500-point x integration leads to: $g_{CM3}^{EQ}=$ 42.930, $g_{ET3}^{EQ}=$ 27.183. (ii) $n_{max}=10$, $y_{max}=70\;Å$ ($y_{max}^{*}=20)$, using 2000-point y integration plus 1000-point x integration leads to $g_{CM3}^{EQ}=$ 42.931, $g_{ET3}^{EQ}=$ 27.183. (iii) $n_{max}=30$, $y_{max}=70\;Å$ ($y_{max}^{*}=20)$, using 2000-point y integration plus 1000-point x integration, lead to: $g_{CM3}^{EQ}=$ 42.932, $g_{ET3}^{EQ}=$ 27.185.

## 4. Results and Discussion

The results reported in this section are complemented with data in the Supplementary Materials.

### 4.1. The Pair Level Structures

**Figure 1:**
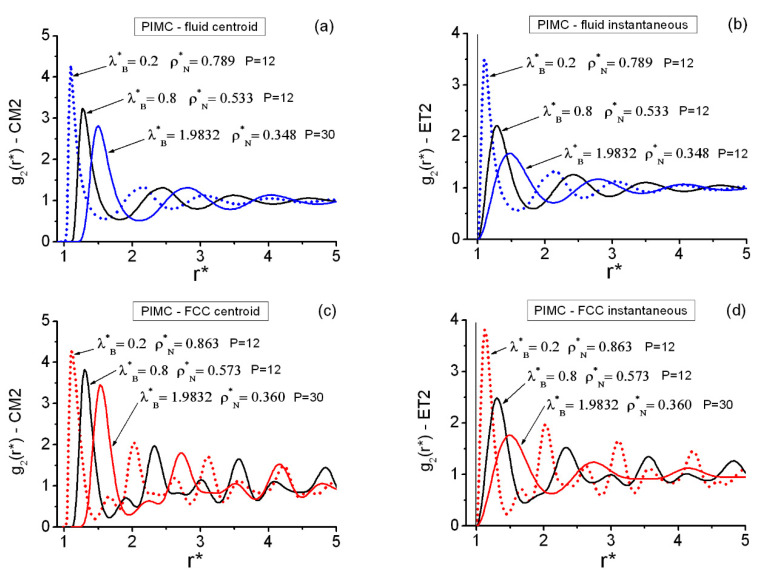
PIMC pair radial correlation functions along the quantum hard spheres (QHS) fluid–FCC (face-centered cubic) solid coexistence line (six state points in Table 1). The arrangement should be clear according to the $\left(\rho_{N}^{*},\;\lambda_{B}^{*}\right)$ values shown. (**a**) Fluid centroid functions. (**b**) Fluid instantaneous functions. (**c**) FCC centroid functions. (**d**) FCC instantaneous functions. The vertical line at $r^{*}=1$ in (**b**,**d**) marks the position of the hard core.

Figure 1 shows representative pair radial correlation functions, centroid, and instantaneous, along the fluid–FCC solid coexistence (see also the Supplementary Materials for more information). The fluid functions (Figure 1a,b) display clear fluid-like features. Analogously, the FCC solid functions (Figure 1c,d) display the expected traits of FCC lattices. General comments on these pair radial functions are (i) the higher order in the solid functions that does not disappear with increasing distances; (ii) the outward shift and smoothing of the features with increasing quantum effects (on the coexistence line analyzed, one has 0.006<γ<2.81); and (iii) the proximity between the locations of the fluid and solid first maxima (also between the dominant second-maximum regions), revealing that the system is ready to effect the change of phase. It is also interesting to note in passing that on the fluid side, the absolute maxima of the pair structures show dependences upon γ that can be fitted in the form $g_{2}\left(Max\right)=a\gamma^{-b}$, the associated linear correlation coefficients $r_{corr.}$ being reasonably good: (a) for the centroid functions, a≅3.0042, b≅0.0687, $r_{corr.}=-0.9982;\;$and (b) for the instantaneous functions, a≅1.8863, b≅0.1233, $r_{corr.}=-0.9999$. Furthermore, the concordance at the pair level between the results in the {r} and the {k} spaces is excellent. The fluid radial functions are fully consistent in particular with the configurational maximal values arising from Equations (14) and (15): the fluid phase maximal values obtained remain $S_{2}^{\left(C\right)}<0.1$. Moreover, Table 3 contains the observed variations in the maximal values of $S_{2}^{\left(C\right)}$ corresponding to the FCC centroid and instantaneous cases. For the current calculations, a representative FCC-set of maximal wave vectors can be defined by their k-integer components: {(−6,6,6),(−6,6,−6,),(6,6,6)}.

**Figure 2:**
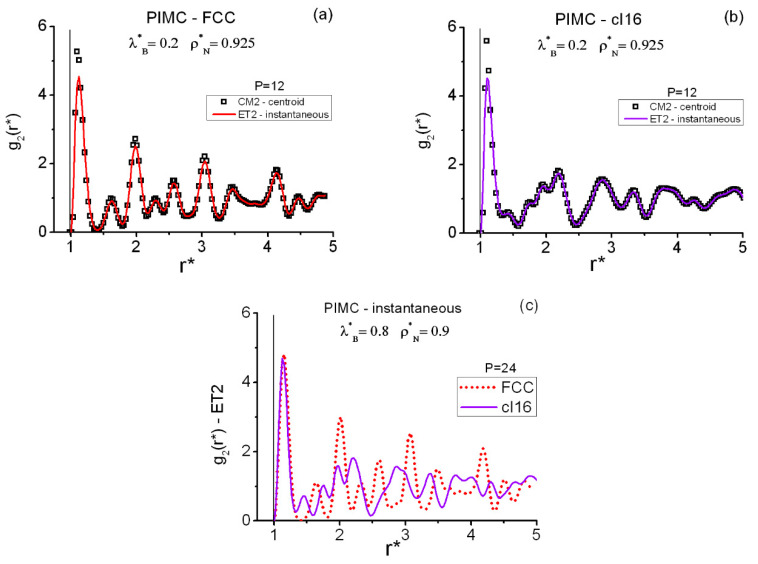
PIMC pair radial correlation functions in the region of moderately high densities for the cubic-based QHS solid phases FCC and cI16. No smoothing of the simulation results has been carried out. The vertical line at $r^{*}=1$ in (**a**–**c**), marks the position of the hard core.

Figure 2 shows the pair radial correlation functions, centroid and instantaneous, of the FCC and *cI16* state points at the moderately high densities $\rho_{N}^{*}=0.9,\;0.925$. There is a sharp contrast between the FCC and *cI16* structures, since the usual coordination shells existing in the highly regular FCC lattice are absent from *cI16.* The most characteristic trait of *cI16* is, perhaps, the presence of a convoluted inner structure, with two conspicuous big dips, for distances below $r^{*}\approx 2.5$. The FCC solid structures (Figure 2a,c) are the “compression” (at constant temperature) of the corresponding FCC structures on the coexistence line. The current *cI16* results (Figure 2b,c) agree feature for feature with the pair structures displayed by the *bcc-qIII* phases in [67]. (Differences between the first peaks are due to the B-spline smoothing carried out in Figure 9 of [67]; see the Suppplementary Material for non-smoothed data). This deserves to be highlighted, since the PIMC-QHS origins of both types of structures are not related: the former *bcc-qIII* phases arose from the evolution of initially perfect *BCC* lattices $\left(N_{S}=432\right)$, while the present (delocalized) *cI16* phases are just the results obtained from the evolution of initially perfect *cI16* lattices $\left(N_{S}=1024\right)$. To complete the foregoing information, Table 3 also contains the variations in the maximal values of the respective *cI16*-configurational $S_{2}^{\left(C\right)}$ structure factors. They are consistent with the behavior reported in [67]. For the current calculations, a representative *cI16*-set of maximal wave vectors can be defined by their *k*-integer components {(−8,8,0),(0,8,−8,),(8,8,0)}.

There is still the further question related to the characterization of cI16 phases via the fractional displacement parameter x. In the quantum case, the delocalization makes this task a three-fold one, since there are three types of distinct structures. Given the current scope, only the centroid and instantaneous x estimates are determined in this work. A convenient way is through the tabulation for perfect cI16 lattices of $\left(x,S_{2}^{\left(C\right)}\left(k_{\max}\right)\right)$, which can be computationally fixed by varying x. Thus, for the interval 0.025≤x≤0.038, using Δx=0.001, the related parabolic least-squares fitting (better than just linear) leads to

(22) $$S_{2}^{\left(C\right)}\left(k_{max}\right)=1.0931-8.269x-108.75x^{2};\;\mathrm{CM}2\;\mathrm{and}\;\mathrm{ET}2,$$

which guarantees absolute errors of orders $\leq 10^{-4}$ in the estimated values of the reference maximal structure factors (for higher precision, the reader is referred to the tabulation in the Supplementary Materials). Note that the higher the x is, the lower the $S_{2}^{\left(C\right)}\left(k_{\max}\right)$ becomes, as expected. In this regard, note that for x=0, which is out of the above interval, one must retrieve the perfect BCC limit $S_{2}^{\left(C\right)}\left(k_{\max}\right)=1$. Consideration of the actual calculated values of the maximal $S_{CM2}^{\left(C\right)}\left(k_{\max}\right)$ and $S_{ET2}^{\left(C\right)}\left(k_{\max}\right)$ in Table 3 yields the cI16 variations: (i) at $\left(\rho_{N}^{*}=0.925,\;\lambda_{B}^{*}=0.2;P=12\right)$, 0.0314≤x≤0.0332 for CM2, and 0.0325≤x≤0.0343 for ET2; and (ii) at $\left(\rho_{N}^{*}=0.9,\;\lambda_{B}^{*}=0.8;P=24\right)$, 0.0277≤x≤0.0282 for CM2, and 0.0323≤x≤0.0328 for ET2. These values show the expected behavior: (a) they are larger for the instantaneous structures; (b) they are consistent with cI16 values reported in the literature [76,77,80]; and (c) the CM2–ET2 differences increase with the quantum effects. Another point to consider here is related to the fact that samples of classical hard spheres can be “squeezed” more than samples of quantum hard spheres, because of the latter’s “preemptive” repulsions. This means that via low temperatures, one can expect QHS–cI16 phases to appear for lower densities than in the classical hard-sphere system $\left(\rho_{N}^{*}\geq 1.1\right)$ [80], which is indeed the case.

### 4.2. Triplets in the Fluid Phase

**Figure 3:**
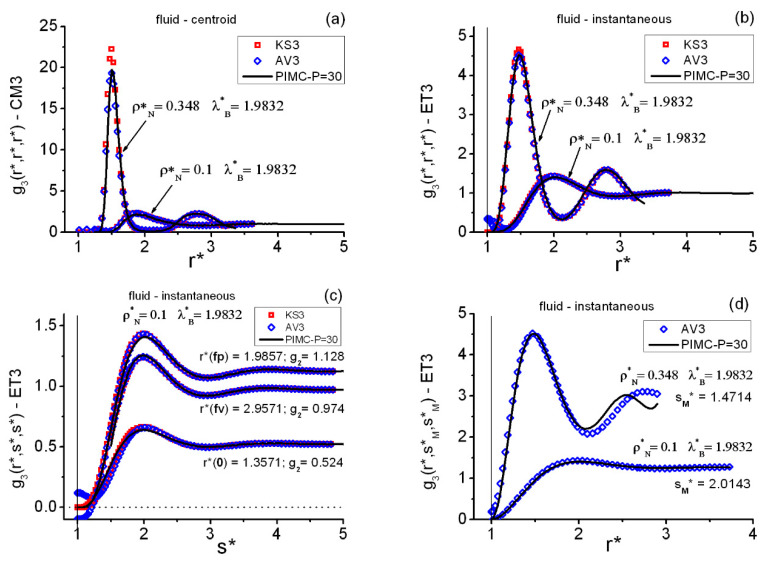
Typical behaviors of the centroid and instantaneous triplet correlations in the QH fluid at two representative state points. KS3 = Kirkwood superposition, Equation (17); AV3 = average closure, Equation (19); PIMC = path integral Monte Carlo. (**a**) Centroid equilateral; (**b**) instantaneous equilateral; (**c**) instantaneous isosceles, with pair $g_{2}\left(r^{*}\right)$ asymptotic values shown (increasing $s^{*})$ at three selected $r^{*}$ (close to the pair first maximum fp, close to the pair first minimum fv, and with 0 being a pair close-range distance); (**d**) $r^{*}$ profiles of the heights in the close vicinity of the first maxima of the instantaneous isosceles correlations ($s_{M}^{*}=$ distance in the close vicinity of where the absolute equilateral maximum appears). The vertical line at $r^{*}=1$ in (**b**–**d**) marks the position of the hard core.

**Figure 4:**
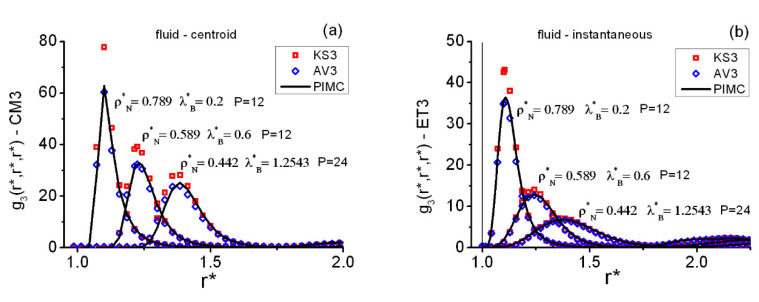
Typical forms of the centroid and the instantaneous equilateral correlations in the QHS fluid at three representative state points on the fluid–FCC coexistence line. Acronyms for methods as in Figure 3. (**a**) Fluid centroid functions. (**b**) Fluid instantaneous functions. The vertical line at $r^{*}=1$ in (**b**) marks the position of the hard core.

**Figure 5:**
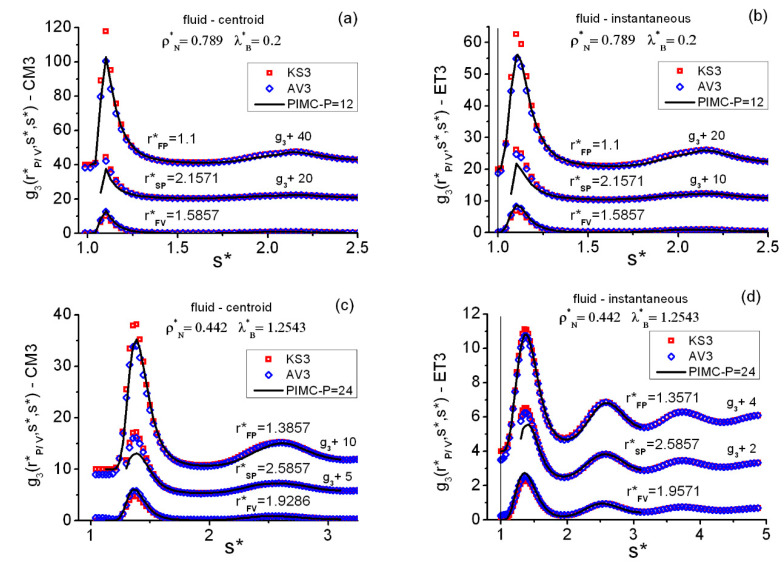
Typical behaviors of the centroid and instantaneous isosceles correlations in the QHS fluid at two representative state points on the fluid–FCC coexistence line. Acronyms for methods as in Figure 3. Results at three especial $r^{*}$ distances of the equilateral correlations very close to the respective: first maximum (FP), first minimum (FV), and second maximum (SP). (**a**) Upper plots shifted by +20 and +40. (**b**) Upper plots shifted by +10 and +20. (**c**) Upper plots shifted by +5 and +10. (**d**) Upper plots shifted by +2 and +4. The vertical line at $r^{*}=1$ in (**b**,**d**) marks the position of the hard core.

Figure 3, Figure 4 and Figure 5 show the main features of the fluid triplet correlations analyzed in this work. Several general trends can be easily identified in Figure 3, which collects results at two state points along the lowest isotherm $\lambda_{B}^{*}=1.9832$. First, as occurred on the pair level, the centroid CM3 features are far more pronounced than those of the instantaneous ET3 case. Second, and associated with the equilateral data, one notes that the first maximum and the first minimum positions of a given $g_{3}\left(r^{*},r^{*},r^{*}\right)$ occur in the close vicinities of the corresponding first maximum and first minimum of the associated $g_{2}\left(r^{*}\right)$ shown in Figure 1. Third, although the closures KS3 and JF3 fail to reproduce the exact PIMC behavior, their average AV3 shows a remarkable performance for both the centroid and the instantaneous correlations. Fourth, as the density increases along isotherms, and when going toward longer distances, AV3 loses predictive power to fit the profiles of the isosceles correlations $g_{3}\left(r^{*},s^{*},s^{*}\right)$. In relation to this, see Figure 3d, where $s^{*}=s_{M}^{*}$ is such that $g_{3}\left(s_{M}^{*},s_{M}^{*},s_{M}^{*}\right)≅$ absolute equilateral maximum.

Finer equilateral facts follow. (i) Figure 3a,b displays explicitly, at state point $\left(\rho_{N}^{*}=0.1,\lambda_{B}^{*}=1.9832\right)$, the equilateral asymptotic behavior $g_{3}\left(r^{*},r^{*},r^{*}\right)\to 1$ with increasing $r^{*}$ for the PIMC centroid and instantaneous correlations. (ii) Figure 3c illustrates the isosceles asymptotic behavior $g_{3}\left(r^{*},s^{*},s^{*}\right)\to g_{2}\left(r^{*}\right)$, when the two $s^{*}$ distances increase. (iii) As seen, the short-range behavior of AV3 is non-correct (due to that of JF3), whereas KS3 behaves properly. (iv) At constant temperature, there is a sharpening and shifting inwards of the structures with increasing density. For example, at $\lambda_{B}^{*}=1.9832$ in the vicinities $\left(r_{FP}^{*}\right)$ of the equilateral first maxima, the $g_{3}^{EQ}=g_{3}\left(r^{*},r^{*},r^{*}\right)$ behave as follows: (1) $\rho_{N}^{*}=0.1$, $\left(r_{FP}^{*}=1.9,g_{CM3}^{EQ}=2.16\right)$ and $\left(r_{FP}^{*}=2,g_{ET3}^{EQ}=1.41\right)$; (2) $\rho_{N}^{*}=0.3$, $\left(r_{FP}^{*}=1.5571,g_{CM3}^{EQ}=12.65\right)$ and $\left(r_{FP}^{*}=1.5429,g_{ET3}^{EQ}=3.54\right)$; and (3) $\rho_{N}^{*}=0.348$, $\left(r_{FP}^{*}=1.5,g_{CM3}^{EQ}=19.74\right)$ and $\left(r_{FP}^{*}=1.4714,g_{ET3}^{EQ}=4.51\right).$ An analogous behavior can be observed at the pair level. (Use σ=3.5 Å and rounding-off to two decimal places to transform the foregoing $r^{*}$ into the actual r of the (M,σ) system utilized in the current calculations, e.g., $r^{*}=1,5571\to r^{*}=5.5\;Å).$

Figure 4 shows the equilateral correlations at three representative state points on the fluid side of the coexistence line. The aforementioned trends of KS3 and AV3, as compared to PIMC, appear again for both types of correlations CM3 (Figure 4a) and ET3 (Figure 4b). In going from higher to lower densities/temperatures on the fluid side, one observes that the larger the quantum effects, the flatter the structural triplet features become.

Table 4 contains the absolute maxima, fixed with quadratic interpolations of the adequate PIMC data, of the fluid equilateral correlations. (See the Supplementary Materials for more related numerical data). Once more, in an attempt to connect the foregoing maximum behaviors with the quantum parameter γ, one notes that simple empirical decay fittings $g_{3}^{EQ}\left(Max\right)=a\gamma^{-b}$ can be found for the centroid and for the instantaneous cases, their associated linear correlation coefficients $r_{corr.}$ being reasonably good. Thus, one finds for the centroid case $a≅23.6702,b≅0.1877,r_{corr.}=-0.9959$ and for the instantaneous case $a≅6.437,b≅0.3449,\;r_{corr.}=-0.9999.$ This general pattern is to be regarded as a reflection of the very same observed at the pair level. Three additional points are worthwhile to mention: (i) the quality of this type of fitting remains comparable if one tries the modification $g_{3}^{EQ}\left(Max\right)=a\gamma^{-b}+c$; (ii) exponential decays, e.g., $g_{3}^{EQ}\left(Max\right)=aexp\left(-b\gamma \right),$ give poor fittings; and (iii) the potential energy discontinuity at $r^{*}=1$ precludes one from retrieving the classical limit at $\lambda_{B}^{*}=0.$ Although there is no apparent physical basis for the empirical γ pattern found, this line of thought might be well worth exploring in future work.

**Figure 6:**
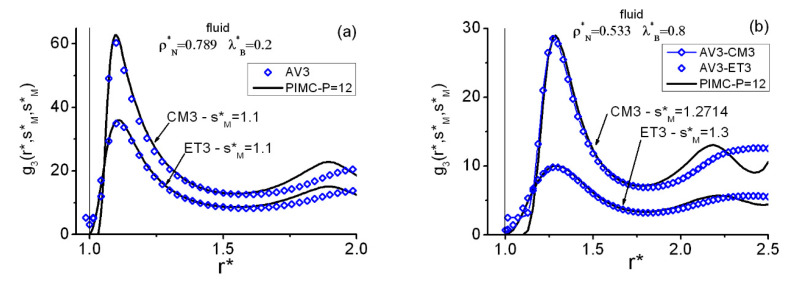
QHS fluid centroid (CM3) and instantaneous (ET3) $r^{*}$ profiles of the heights in the close vicinities of the first peaks of the isosceles correlations at two selected state points on the fluid–FCC coexistence line. (**a**) Fluid functions at $\left(\rho_{N}^{*}=0.789,\;\lambda_{B}^{*}=0.2\right)$. (**b**) Fluid functions at $(\rho_{N}^{*}=0.533,\;\lambda_{B}^{*}=0.8).$ $s_{M}^{*}=$ distance in the close vicinity of the absolute maximum of the equilateral correlations. Acronyms for methods as in Figure 3. The vertical line at $r^{*}=1$ marks the position of the hard core.

Figure 5 contains a quick description of the isosceles correlations $g_{3}\left(r^{*},s^{*},s^{*}\right)$ at two representative fluid state points, for the centroids CM3 in panels (a)–(c) and for the instantaneous ET3 in panels (b)–(d). Three especial $r^{*}$ distances are selected from the $g_{3}\left(r^{*},r^{*},r^{*}\right)$ information obtained at each state point, namely $r_{FP}^{*}$, $r_{FV}^{*}$, and $r_{SP}^{*}$, which are positions in the close vicinities of the equilateral maxima and minima: first maximum (FP), first minimum (FV), and second maximum (SP), respectively. Apart from the expected AV3 unphysical behavior for $r^{*}\leq 1$, the good overall performance of AV3 is certainly surprising. Two weak points are to be remarked. First, the AV3 (and KS3) behavior for low $s^{*}$ distances, $1<s^{*}<1.5$, when $r^{*}$ increases: for example, at $r_{SP}^{*}$ where the closure maxima are overestimated. (This is directly related to the AV3 trend displayed by the upper profile plot in Figure 3d). Second, Figure 6 shows a detailed image of the isosceles deterioration of the PIMC–AV3 agreement with increasing densities, the worse results for centroids being a consequence of this key fact (centroids mimic a fluid at a higher density than the actual one).

### 4.3. FCC triplets on the Fluid–Solid Coexistence Line

**Figure 7:**
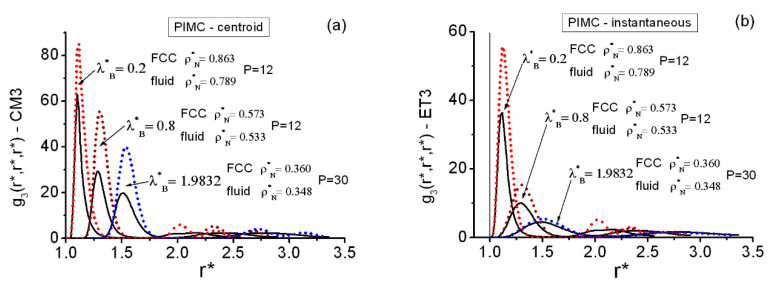
Comparison between PIMC equilateral structures on both sides of the fluid–FCC coexistence line at selected state points. (**a**) Centroid functions. (**b**) Instantaneous functions. The vertical line at $r^{*}=1$ in (**b**) marks the position of the hard core.

**Figure 8:**
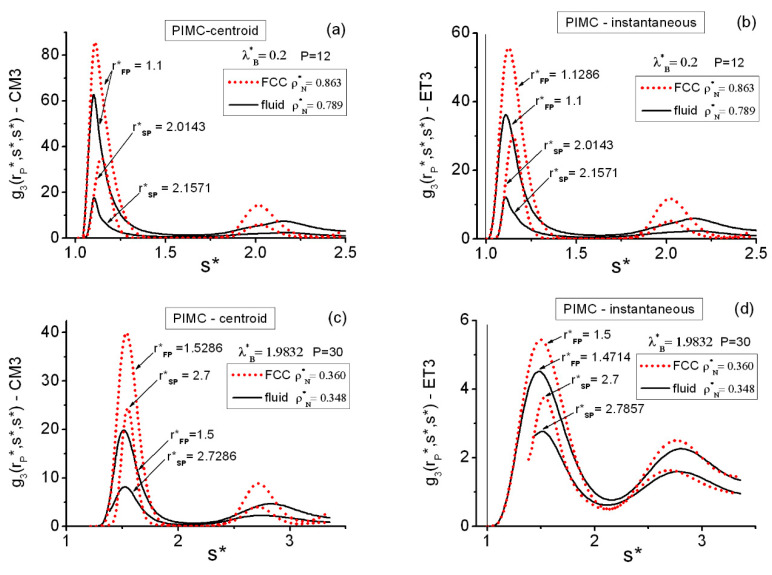
Comparison of PIMC isosceles triplet structures on both sides of the fluid–FCC coexistence line at selected state points and $r_{FP}^{*}$ and $r_{SP}^{*}$ slices. These $r^{*}$ are very close to the first (FP) and second (SP) maxima of the corresponding equilateral structures. (**a**) Centroid functions at $\lambda_{B}^{*}=0.2.$ (b) Instantaneous functions at $\lambda_{B}^{*}=0.2.$ (c) Centroid functions at $\lambda_{B}^{*}=1.9832.$ (d) Instantaneous functions at $\lambda_{B}^{*}=1.9832.$ The vertical line at $r^{*}=1$ in (**b**,**d**) marks the position of the hard core.

Table 4 and Figure 7 and Figure 8 show selected results for the PIMC equilateral and isosceles correlations of FCC state points on the solid side of the fluid–FCC coexistence line, within the approximations obtainable from Equations (7) and (8). For visualization purposes, the associated PIMC fluid results are also incorporated into these figures.

A general idea can be obtained by observing Table 4. The fluid and solid absolute maximum positions are close to one another, and the structures are shifted outwards with increasing quantum effects. Moreover, higher $g_{3}$ values appear on the solid side (e.g., at $\lambda_{B}^{*}=0.2,\;\approx +39\%$ for CM3, and ≈+51% for ET3). This trend is far more pronounced for the centroid correlations, the ratio increasing monotonically with $\lambda_{B}^{*}$ (e.g., at $\lambda_{B}^{*}=1.9832,\;\approx +102\%$ for CM3). However, for the instantaneous ET3 correlations, such a ratio is not monotonic; it goes through a maximum (at $\lambda_{B}^{*}=0.4,\;\approx +62\%$) and then falls monotonically (at $\lambda_{B}^{*}=1.9832,\;\approx +20\%$). These behaviors can be ascribed to the two effects present on the coexistence line. On the one hand, there is the decreasing density, which contributes to diminishing the structural features. On the other hand, there is the increasing delocalization with $\lambda_{B}^{*}$, which makes PI structures become more and more smeared out, the instantaneous case being always much more sensitive to this. As regards the question of finding a γ−fitting of the solid equilateral absolute maxima, the situation is less clear than on the fluid side (γ is slightly higher on the solid side). Although one can obtain reasonable dependences $g_{3}^{EQ}\left(Max\right)=a\gamma^{-b}$ $\left(r_{corr.}<-0.991\right)$, on closer inspection, these fittings cannot cope with the apparent inflection in 0.13<γ<0.3 (or in $0.6<\lambda_{B}^{*}<0.8)$ for centroids $g_{CM3}^{EQ}\left(Max\right)$, nor with the large discrepancies for low γ between the original and the estimated instantaneous values $g_{ET3}^{EQ}\left(Max\right)$.

In Figure 7, one observes that the equilateral FCC and fluid $g_{3}\left(r^{*},r^{*},r^{*}\right)$ patterns are qualitatively similar within the first maximum regions. It is also noticeable that the FCC state points develop easily identifiable peak structures with increasing distances $\left(r^{*}≳2\right).$ The main two maxima of the FCC equilateral triplets can be put into direct correspondence with the main two maxima obtained at the FCC pair level (Figure 1), since they appear located close to one another.

The FCC $g_{3}\left(r^{*},r^{*},r^{*}\right)$ display deep first valleys, almost at the zero-ground level, appearing for both the centroid and the instantaneous structures, e.g., for centroids and $\rho_{N}^{*}=0.573$, the region in Figure 7 located in $1.6≲r^{*}≲2.1$. In general, this feature is far more pronounced in the centroid structures than in the instantaneous structures and is consistently shifted outwards with increasing quantum effects. If comparison with Figure 1c,d is made, one notes that this triplet region corresponds to the FCC pair region where the smallest maximum shows up. (Such region fades away with increasing quantum effects in the instantaneous case, Figure 1d). To get a feeling of the depth of these valleys, it seems worthwhile to quote some significant results: (a) at $\left(\rho_{N}^{*}=0.863,\lambda_{B}^{*}=0.2\right)$, within the range $1.4143\leq r^{*}\leq 1.8143$, the equilateral centroid and instantaneous values remain $g_{CM3}^{EQ}≲0.07$ and $g_{ET3}^{EQ}≲0.09$, respectively; (b) at $\left(\rho_{N}^{*}=0.573,\lambda_{B}^{*}=0.8\right)$, within the range $1.6143\leq r^{*}\leq 2.0714$, the equilateral centroid values remain $g_{CM3}^{EQ}≲0.1$, whereas the equilateral instantaneous values reach the same upper bound $g_{ET3}^{EQ}≲0.1$ within the narrower range $1.6714\leq r^{*}\leq 1.9571$; and (c) at $\left(\rho_{N}^{*}=0.360,\lambda_{B}^{*}=1.9832\right)$, within the range $1.9\leq r^{*}\leq 2.3571$, the equilateral centroid values remain $g_{CM3}^{EQ}≲0.08$, whereas the equilateral instantaneous values $g_{ET3}^{EQ}$ do not go below 0.15 within their related first valley. (See the Supplementary Materials for more data on the coexistence line).

In addition, Figure 8 contains typical isosceles $g_{3}\left(r^{*},s^{*},s^{*}\right)$ behaviors of the fluid and the FCC solid at the lowest $\left(\lambda_{B}^{*}=0.2\right)$ and the highest $\left(\lambda_{B}^{*}=1.9832\right)$ de Broglie wavelengths. These graphs display significant $r^{*}-$slices (i.e., at $r_{FP}^{*}$ and $r_{SP}^{*}$) of the tabulated functions in the close vicinities of the corresponding first (FP) and second (SP) maxima of the equilateral correlations. The parallels between the triplets of the solid and fluid phases coexisting at equilibrium are manifest once more.

### 4.4. Triplets in the FCC and cI16 Denser Solid Structures

**Figure 9:**
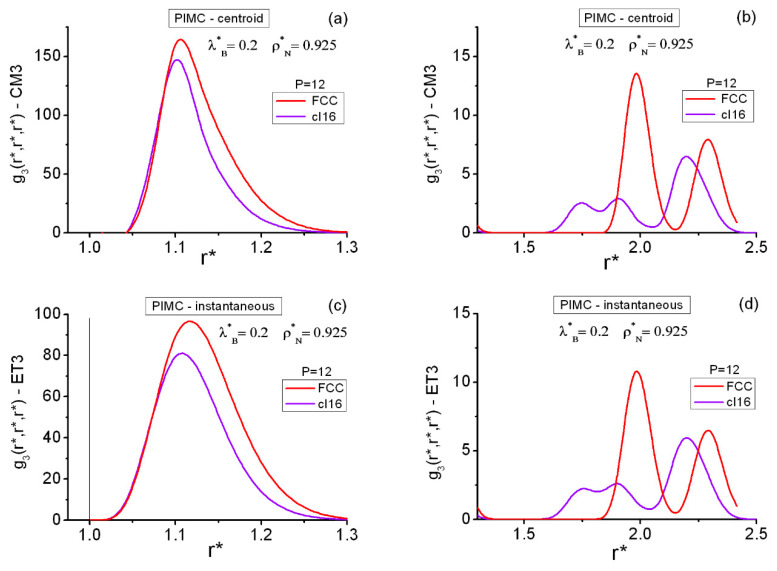
PIMC results for the FCC and cI16 equilateral structures in the region of moderately high densities $\rho_{N}^{*}$. The graphs are split horizontally into two parts to avoid the flat $g_{3}$ range of distances and show the secondary maximum regions on a visible scale. (**a**) Centroid functions in the short-distance range. (**b**) Centroid functions in the medium-distance range. (**c**) Instantaneous functions in the short-distance range. (**d**) Instantaneous functions in the medium-distance range. The vertical line at $r^{*}=1$ in (**c**) marks the position of the hard core.

Figure 9 and Table 5 contain equilateral PIMC results for the FCC and *cI16* state points in the region of moderately high densities $\left(\rho_{N}^{*}=0.9,0.925\right)$. The centroid CM3 and the instantaneous ET3 correlation results, with P=12 for both lattices at $\left(\rho_{N}^{*}=0.925,\;\lambda_{B}^{*}=0.2\right)$, are P converged (Table 2). At $(\rho_{N}^{*}=0.9,\;\lambda_{B}^{*}=0.8),$ convergence for the instantaneous correlations with P=36 is guaranteed (practical convergence already occurs with P=24), whereas for the centroid correlations, there is still room for further improvement. Nevertheless, the centroid results obtained with P=36 are expected to capture well the related global features. This contrasts with the more rapid P convergence for centroids at the pair level.

In Figure 9, the equilateral correlations of the FCC and *cI16* state points at $\left(\rho_{N}^{*}=0.925,\;\lambda_{B}^{*}=0.2\right)$ are considered within $r^{*}<2.5$. Three well-defined features can be seen in each case, and they can be put into correspondence with the results obtained at the related distances on the pair level (Figure 2). Thus, three separated maxima arise from the triplet FCC calculations (as occurred on the coexistence line). However, four maxima arise from the triplet *cI16* calculations, with the second and third forming an overlapping structure. This reminds one of the characteristic shallow split showing up past the first maximum in the $g_{2}\left(r^{*}\right)$ of amorphous systems [99]. Moreover, the FCC features are more pronounced than those of *cI16,* as was to be expected. In addition, for $1.5<r^{*}<2.5$, *cI16* and FCC are somewhat complementary regarding the positions of their peaks. One observes the clear quantitative differences between the centroid CM3 and the instantaneous ET3 results. The patterns of the salient features shown in Table 5 for the two density–temperature conditions are fully consistent with each other and with the underlying pair information (Figure 2).

A closer inspection of the equilateral flat regions between the first and the second maxima may be worth carrying out. The following results correspond to the discretizations: (i) P=24 at $\left(\rho_{N}^{*}=0.925,\;\lambda_{B}^{*}=0.2\right)$, although P=12 results are not significantly different; and (ii) P=36 at $\left(\rho_{N}^{*}=0.9,\;\lambda_{B}^{*}=0.8\right)$.

(a) As regards the FCC results, these regions are related to those found on the coexistence line, but now the behavior is much more extreme: the zero-ground level is effectively reached. At $\left(\rho_{N}^{*}=0.925,\;\lambda_{B}^{*}=0.2\right)$, centroid values $g_{CM3}^{EQ}\equiv 0$ are obtained within $1.4714\leq r^{*}\leq 1.6429$, while instantaneous values $g_{ET3}^{EQ}\equiv 0$ are within $1.5286\leq r^{*}\leq 1.5857$. Moreover, at $\left(\rho_{N}^{*}=0.9,\;\lambda_{B}^{*}=0.8\right)$, centroid values $g_{CM3}^{EQ}\equiv 0$ are obtained within $1.2714\leq r^{*}\leq 1.9$, while instantaneous values $g_{ET3}^{EQ}\equiv 0$ are within $1.4714\leq r^{*}\leq 1.7$.
(b) The situation of cI16 is less severe, although with increasing quantum effects, some of the previous traits also arise. Thus, at $\left(\rho_{N}^{*}=0.925,\;\lambda_{B}^{*}=0.2\right)$, centroid values remain $g_{CM3}^{EQ}≲0.04$ within $1.3286\leq r^{*}\leq 1.5857$, with $g_{CM3}^{EQ}\equiv 0$ only for $1.44\leq r^{*}\leq 1.47$, while the instantaneous values are above zero in that latter region $\left(0<g_{ET3}^{EQ}≲0.08\right)$. At $\left(\rho_{N}^{*}=0.9,\;\lambda_{B}^{*}=0.8\right)$, centroid values $g_{CM3}^{EQ}\equiv 0$ appear within $1.2429\leq r^{*}\leq 1.6429$, while instantaneous values $g_{ET3}^{EQ}\equiv 0$ do only for $1.44\leq r^{*}\leq 1.47$.

The solid triplet flat regions arise from the combination of the role of the QHS interactions and the unavailability of space due to the variations in $\rho_{N}^{*}$ and $\lambda_{B}^{*}$. As a result, the solid equilateral structures analyzed turn out to be simpler than their pair radial counterparts (Figure 2), which is especially true of the *cI16* lattice. Use of this fact might find application to characterizing irregular solid structures and/or monitoring their formation. (See the Supplementary Materials for more information on these structures). Another observation is related to the order shown by these two lattices. FCC appears as more ordered than *cI16,* which is clear from the respective regularities in their pair and triplet correlation functions and also from the maximal configurational structure factors (Table 3). Therefore, under the same$\;(\rho_{N}^{*},\lambda_{B}^{*})$ conditions, FCC entropies (and free energies) [67] must be lower than those of *cI16*, the determination of these properties being possible via the Einstein crystal quantum technique [66,67].

## 5. Conclusions

This article has analyzed several real space triplet correlation issues in the PI–QHS system under conditions in which quantum exchange can be neglected. Triplet PI centroid and instantaneous correlations (equilateral and isosceles) in significant fluid and FCC–solid-state points have been studied. Furthermore, the positive identification of the formerly denoted bcc–qIII solid phases [67,78] with proper quantum cI16 solid phases has been achieved by utilizing information at the pair level (radial structures and maximal structure factor values). Triplet calculations have also been carried out at two cI16 state points. The results lead to the following conclusions.

(1) Fluid phase and the use of closures.
  (a) The centroid results display far more structured triplet functions than the instantaneous results. These structures tend to be shifted outwards with increasing $\lambda_{B}^{*}$ (delocalization) and inwards with increasing $\rho_{N}^{*}$ (localization).
  (b) From the comparison between PIMC and the closure results, one concludes that the role of pair correlations in shaping triplet structures is more relevant in the quantum domain than previously thought. The combined use of KS3 (for short range) plus AV3 = (KS3+JF3)/2 (beyond short range), although not exact, is found to be a useful and simple choice to understand the related main {r} triplet features, either centroid or instantaneous, of fluids with quantum diffraction effects.
  (c) The AV3 success appears to be linked with the fact that this closure adopts the form of a “complete” $h_{2}\;$expansion truncated to first order in the density, which includes explicitly the triple-$h_{2}$ product absent from JF3. Given that along isotherms, AV3 deteriorates with increasing distances as the fluid–solid coexistence is approached, improvements on AV3 may be of interest and should incorporate at least second-order density terms in the $h_{2}\;$expansion.
  (d) The foregoing finding extends the previous results obtained in [17] for liquid para-hydrogen and liquid neon, since the current study has involved a purely repulsive interparticle potential. Therefore, applications of an improved AV3 (supplemented with KS3 as said above) might be expected to provide reliable pictures of what is behind triplet correlations in fluid helium over a wide range of conditions [4,15].
(2) The fluid–FCC solid coexistence line.
  (a) There is a close correspondence between the positions of the main structural features at short range of both phases at equilibrium, not only at the pair level but also at the triplet level. Such a phase correspondence between triplet positions appears in both the equilateral and the isosceles correlations. These are clear signs of the system readiness to undergo the phase transition.
  (b) The triplet features are far more pronounced on the solid side. In addition, the centroid features are always sharper than those of their instantaneous counterparts (e.g., more elevated peak regions and lower valley regions for centroids).
(3) On the fluid side, the absolute maxima of the pair and the triplet-equilateral correlations, centroid and instantaneous, appear to follow empirical behaviors that depend on the quantum parameter $\gamma =\rho_{N}^{*}\lambda_{B}^{*3}$ in the general form $g_{3}^{EQ}\left(\mathrm{Max}\right)=a\gamma^{-b}$. For systems in which repulsive particle interactions dominate, this might be a further structural signature of the fluid phase on the quantum crystallization line [17,18,49], and it deserves further examination.
(4) FCC and cI16 solid phases.
  (a) FCC state points show a significantly higher order than their cI16 counterparts, at the same density–temperature conditions, which can be ascribed to the openness of the cI16 lattice. This is observed for the two structures, centroid and instantaneous, in all the forms computed $\left(g_{2},\;g_{3},\;S_{2}^{\left(C\right)}\right)$. Roughly speaking, at a given state point, using the maximal values of the configurational structure factors, one finds that $S_{CM2}^{\left(C\right)}\left(FCC\right)/S_{CM2}^{\left(C\right)}\left(\mathrm{cI}16\right)\approx S_{ET2}^{\left(C\right)}\left(\mathrm{FCC}\right)/S_{ET2}^{\left(C\right)}\left(\mathrm{cI}16\right)$. FCC entropies must certainly be lower than their cI16 counterparts, and it is tempting to explore the relationships between the solid entropy and the values of the quantum structure factors in future work.
  (b) Within the short–medium range of distances (i.e., $1<r^{*}<2.5)$ the equilateral functions adopt shapes simpler than the pair radial functions. This effect turns out to be much more remarkable for cI16 state points, which show quite a convoluted peak/valley behavior. Accordingly, for the purposes of monitoring the onset of crystallization and/or characterizing irregular solid phases in general, triplet centroid information may advantageously complement the usual pair level information.
  (c) PIMC calculations of solid centroid triplet structures converge slowly with increasing quantum effects, which contrasts with the more rapid convergence of the centroid pair calculations. This fact should be kept in mind when studying centroid triplets in high-density solid phases at low temperatures.
(5) Finally, one must dwell a little more on the (mechanically stable) QHS–cI16 phase that, as is shown in this work and [67], arises for lower densities than in the classical case. Once the question of its appearance from the PIMC evolution of perfect BCC lattices has been settled, there are no symmetry problems related to the calculations of cI16 free energies [67]. The selection of an appropriate reference system (Einstein crystal [66,67]) can be well defined now [80], and the way to computational studies of stability is open. Although there is every reason for believing that, as in the classical case [80], quantum–cI16 is metastable with respect to FCC (or to HCP = hexagonal close-packed) at low temperatures, due to the cI16 higher energies and pressures [67], the assessment of such behavior seems highly valuable. In this connection, one notes the potential QHS-cI16 relations to (i) the high-pressure solid–solid transitions in alkali metals at low temperatures and (ii) the special responses to external fields that these solid structures, which are less tight than FCC (or HCP), might exhibit.

There is work in progress to tackle the issues raised above and to identify some essential facts associated with quantum condensed matter triplets in the real and the Fourier spaces.

## Figures and Tables

**Table 1:** Fluid and solid-state points of the hard-sphere system studied. Reduced densities $\rho_{N}^{*}$, reduced de Broglie wavelengths $\lambda_{B}^{*}$, path integral Monte Carlo (PIMC) sample size $N_{S}\times P.$

I.1. PHASE TRANSITION ^1^				
	FLUID PHASE		FCC PHASE	
$\lambda_{B}^{*}$	$\rho_{N}^{*}$	$N_{S}\times P$	$\rho_{N}^{*}$	$N_{S}\times P$
0.2	0.789	864 × 12	0.863	864 × 12
0.4	0.672	864 × 12	0.731	864 × 12
0.6	0.589	864 × 12	0.635	864 × 12
0.8	0.533	864 × 12 864 × 24	0.573	864 × 12 864 ×18
1.2543	0.442	864 × 24	0.465	864 × 24
1.9832	0.348	864 × 30 864 × 40	0.360	864 × 30
**I.2. FLUID PHASE**				
1.9832	0.1	864 × 30	---------	---------
1.9832	0.3	864 × 30	---------	---------
**I.3 SOLID PHASES**				
	**cI16 PHASE**		**FCC PHASE**	
0.2	0.925	1024 × 12 1024 × 24	0.925	864 × 12 864 × 24
0.8	0.900	1024 × 12 1024 × 24 1024 ×36	0.900	864 × 12 864 × 24 864 ×36

^1^ Phase transition de Broglie wavelengths and densities fixed in [66,67].

**Table 2:** Selected PIMC convergence features. Centroid (CM3) and instantaneous (ET3) first peaks (FP) in the close vicinities of the equilateral absolute maxima. Number in parentheses are one-standard deviation affecting the last digit(s) ^1^.

$\lambda_{B}^{*}$	$\rho_{N}^{*}$	$N_{S}\times P$	$r_{FP-CM3}^{*}$	$g_{CM3}^{EQ}$	$r_{FP-ET3}^{*}$	$g_{ET3}^{EQ}$
**FLUID PHASE (fluid–FCC coexistence line)**						
1.9832	0.348	864 × 30 864 ×40	1.5	19.7 (5) 19.9 (5)	1.4714	4.51 (2) 4.53 (3)
**FCC PHASE (fluid–FCC coexistence line)**						
0.8	0.573	864 × 12 864 × 18	1.3	55.6 (4) 56.3 (5)	1.3	15.4 (0) 15.4 (0)
**FCC PHASE**						
0.2	0.925	864 × 12 864 × 24	1.1	160.1 (7) 160.0 (15)	1.1286	93.4 (2) 93.1 (3)
0.8	0.9	864 × 12 864 × 24 864 × 36	1.1571	2339 (7) 3028 (16) 3177 (24)	1.1571	112.2 (1) 114.7 (2) 114.1 (2)
**cI16 PHASE**						
0.8	0.9	1024 × 12 1024 × 24 1024 × 36	1.1571	1469 (11) 1233 (10) 1334 (14)	1.1286	158.0 (2) 96.7 (2) 97.8 (3)

^1^19.7(5)≡19.7±0.5; 160.0 (15) ≡160.0±1.5;2339(7)≡2339±7.

**Table 3:** Solid phase variations in the maximal values of the centroid (CM2) Equation (14) and instantaneous (ET2) Equation (15) configurational structure factors at the pair level fixed with PIMC.

FCC PHASE on the Coexistence Line				
$\lambda_{B}^{*}$	$\rho_{N}^{*}$	$N_{S}\times P$	$S_{CM2}^{\left(C\right)}\left(k_{max}\right)$	$S_{ET2}^{\left(C\right)}\left(k_{max}\right)$
0.2	0.863	864 × 12	0.803–0.764	0.786–0.748
0.4	0.731	864 × 12	0.791–0.751	0.738–0.702
0.6	0.635	864 × 12	0.778–0.738	0.686–0.649
0.8	0.573	864 × 12	0.784–0.752	0.643–0.613
1.2543	0.465	864 × 24	0.771–0.732	0.532–0.503
1.9832	0.360	864 × 30	0.743–0.691	0.393–0.356
**FCC PHASE**				
0.2	0.925	864 × 12 864 × 24	0.886–0.866 0.883–0.865	0.867–0.849 0.864–0.847
0.8	0.9	864 × 12 864 × 24 864 × 36	0.986–0.984 0.989–0.987 0.989–0.988	0.898–0.894 0.902–0.898 0.901–0.900
**cI16 PHASE**				
0.2	0.925	1024 × 12 1024 × 24	0.726–0.698 0.732–0.705	0.710–0.682 0.717–0.689
0.8	0.9	1024 × 12 1024 × 24 1024 × 36	0.793–0.784 0.781–0.774 0.777–0.771	0.741–0.732 0.712–0.705 0.708–0.702

**Table 4:** Absolute maxima of the PIMC equilateral correlations $g_{3}^{EQ}=g_{3}\left(r^{*},r^{*},r^{*}\right)$ on the fluid and the FCC sides of the QHS coexistence line. Discretizations at $\lambda_{B}^{*}=0.8$ and 1.9832: P=12 and 30, respectively. $r^{*}=r/\sigma$. Four decimals shown in $g_{3}^{EQ}$ to avoid rounding-off errors.

	FLUID–CENTROID–			FCC–CENTROID–		
$\lambda_{B}^{*}$	$\rho_{N}^{*}$	$r_{Max}^{*}$	$g_{CM3}^{EQ}$	$\rho_{N}^{*}$	$r_{Max}^{*}$	$g_{CM3}^{EQ}$
0.2	0.789	1.1029	63.1089	0.863	1.1097	87.4597
0.4	0.672	1.1690	42.9419	0.731	1.1867	66.8718
0.6	0.589	1.2313	32.7749	0.635	1.2504	56.1959
0.8	0.533	1.2841	29.4898	0.573	1.3042	55.7136
1.2543	0.442	1.3841	25.1954	0.465	1.4051	48.7081
1.9832	0.348	1.5096	19.8989	0.360	1.5360	40.1005
	**FLUID–INSTANTANEOUS–**			**FCC–INSTANTANEOUS–**		
$\lambda_{B}^{*}$	$\rho_{N}^{*}$	$r_{Max}^{*}$	$g_{ET3}^{EQ}$	$\rho_{N}^{*}$	$r_{Max}^{*}$	$g_{ET3}^{EQ}$
0.2	0.789	1.1101	36.7840	0.863	1.1255	55.7274
0.4	0.672	1.1832	19.2805	0.731	1.1990	31.3301
0.6	0.589	1.2419	12.9259	0.635	1.2584	20.1514
0.8	0.533	1.2890	10.0773	0.573	1.3029	15.4292
1.2543	0.442	1.3752	6.8439	0.465	1.3880	9.1774
1.9832	0.348	1.4820	4.5227	0.360	1.4947	5.4411

**Table 5:** Average salient features of the cI16 and FCC equilateral centroid (CM3) and instantaneous (ET3) correlations $g_{3}^{EQ}=g_{3}\left(r^{*},r^{*},r^{*}\right)$. PIMC results in the close vicinities of the maxima and minima. Maxima: first FP, second SP, third TP, fourth F4P. Minima: first FV, second SV, third TV.

	$\left(\rho_{N}^{*}=0.925,\;\lambda_{B}^{*}=0.2\right)$			
	$\mathrm{cI}16-\left(r^{*},g_{3}^{EQ}\right)-PIMC$ −P=12		$\mathrm{FCC}-\left(r^{*},g_{3}^{EQ}\right)-PIMC$ −P=12	
	CM3	ET3	CM3	ET3
FP	(1.1, 146.80)	(1.1, 79.46)	(1.1, 160.07)	(1.1286, 93.38)
FV	(1.4714, 0)	(1.4714, 4 × 10^−5^)	(1.5571, 0)	(1.5571, 0)
SP	(1.7571, 2.52)	(1.7571, 2.25)	(1.9857, 13.53)	(1.9857, 10.80)
SV	(1.8143, 1.87)	(1.8143, 2.02)	(2.1571, 0.31)	(2.1571, 0.51)
TP	(1.9, 2.91)	(1.9, 2.61)	(2.3, 7.82)	(2.3, 6.42)
TV	(2.0429, 0.51)	(2.0429, 0.75)		
F4P	(2.1857, 6.40)	(2.1857, 5.86)		
	$\left(\rho_{N}^{*}=0.9,\lambda_{B}^{*}=0.8\right)$			
	**cI16-** $\left(r^{*},g_{3}^{EQ}\right)-PIMC$ **-** P=36		**FCC-** $\left(r^{*},g_{3}^{EQ}\right)-PIMC$ **-** P=36	
	**CM3**	**ET3**	**CM3**	**ET3**
FP	(1.1571, 1334)	(1.1286, 97.82)	(1.1571, 3177)	(1.1571, 114.08)
FV	(1.4429, 0)	(1.4571, 0)	(1.5857, 0)	(1.5857, 0)
SP	(1.7571, 83.72)	(1.7571, 4)	(2.0143, 425.91)	(2.0143, 18.70)
SV	(1.8714, 0)	(1.8429, 0.92)	(2.1429, 0)	(2.1714, 0.245)
TP	(1.9571, 72.32)	(1.9571, 3.12)	(2.3286, 260.89)	(2.3286, 11.90)
TV	(2.1, 0)	(2.0714, 0.43)		
F4P ^1^	(2.3, 0.44)	(2.2143, 5.68)		

^1^ There is a cI16 small bump at $r^{*}=2.2143$, $g_{\mathrm{CM}3}^{EQ}=0.01\;\left(P=12\right),\;0.18\;\left(P=24\right),\;0.14\;\left(P=36\right)$.

## Supplementary Materials

The following are available online at https://www.mdpi.com/1099-4300/22/12/1338/s1. SupMat2_Entropy.zip. File1: LMS_SupMat_20S_X1.pdf, contents: PIMC-$g_{2}\left(r\right)$ for bcc–qIII and cI16, PIMC convergence, Structure factors values for perfect cI16 lattices, PIMC salient features on the fluid–FCC coexistence line, complete set of fluid pair radial functions (Figure), cI16 and FCC isosceles correlations, comparison between FCC and cI16 equilateral results (Figure). Triplet functions at $(\rho_{N}^{*}=0.672,\;\lambda_{B}^{*}=0.4):$ File2: LMS_SupMat_20S_zgcm3_l4.r672 (centroids), and File3: LMS_SupMat_20S_zget3_l4.r672 (instantaneous).
