# Supplementary material for: Real Space Triplets in Quantum Condensed Matter: Numerical Experiments Using Path Integrals, Closures, and Hard Spheres
Source: Entropy (Basel). 2020 Nov 25;22(12):1338. doi: 10.3390/e22121338 (PMC7759805; doi:10.3390/e22121338)
Supplement: Supplementary file 1 [file entropy-22-01338-s001.zip › s/LMS_SupMat_20S_X1.pdf]

Supplementary Material

Real Space Triplets in Quantum Condensed Matter: Numerical Experiments Using Path Integrals, Closures, and Hard Spheres

Luis M. Sesé

Departamento de Ciencias y Técnicas Fisicoquímicas, Facultad de Ciencias, Universidad Nacional de Educación a Distancia, Avda. Esparta s/n, 28232 Las Rozas, Madrid, Spain

Contents:

Table SM.I

PIMC numerical results for the QHS phases bcc-qIII (Ref. [67]) and cI16 (this work) at state point ($\rho_N^* = 0.925, \lambda_B^* = 0.2$).

Table SM.II

PIMC numerical results for the QHS phases bcc-qIII (Ref. [67]) and cI16 (this work) at state point ($\rho_N^* = 0.9, \lambda_B^* = 0.8$).

Table SM.III

PIMC convergence details.

Table SM.IV

Maximal pair structure factor values vs. x-parameter for perfect cI16 lattices.

Table SM.V

Salient equilateral features of the PIMC triplet correlation functions on the fluid side of the fluid-solid coexistence line.

Table SM.VI

Salient equilateral features of the PIMC triplet correlation functions on the FCC solid side of the fluid-solid coexistence line.

Table SM.VII

cI16 isosceles results in the region of moderately high densities.

Table SM.VIII

FCC isosceles results in the region of moderately high densities.

Figure SM 1

Complete sets of the pair radial correlations on the fluid-FCC coexistence line.

Figure SM 2

FCC and cI16 equilateral results in the region of moderately high densities.

Table SM.I. Selected values of the PIMC pair structures (no B-spline smoothing) for the QHS state points at $(\rho_N^* = 0.925, \lambda_B^* = 0.2)$ of the bcc-qIII (Ref. [67]) and cI16 (this work) solids. Distances r in Å, corresponding to the model $M = 28.0134$ amu, $\sigma = 3.5$ Å. Sample sizes $(N_S \times P)$ shown. Most g_2 values rounded off to two decimals.

Centroid - $g_{CM2}(r)$			Instantaneous - $g_{ET2}(r)$		
r/Å	bcc-qIII (432×12)	cI16 (1024×12)	r/Å	bcc-qIII (432×12)	cI16 (1024×12)
3.45	0	0	3.5	0	0
3.65	0.59	0.59	3.595	0.84	0.84
3.85	5.59	5.60	3.65	1.88	1.88
4.15	2.56	2.57	3.85	4.51	4.52
4.65	0.52	0.51	4.75	0.55	0.54
4.95	0.59	0.60	4.95	0.57	0.58
5.55	0.20	0.20	5.55	0.25	0.25
6.15	0.90	0.90	6.85	1.36	1.37
6.35	0.85	0.83	7.15	1.28	1.27
6.85	1.40	1.41	7.65	1.75	1.75
7.15	1.24	1.23	8.65	0.25	0.25
7.65	1.82	1.82	9.95	1.55	1.54
8.65	0.23	0.22	11.05	0.77	0.76
9.95	1.58	1.57	11.65	1.20	1.21
11.05	0.74	0.73	12.35	0.50	0.50
11.65	1.24	1.25	13.35	1.31	1.30
12.35	0.46	0.46			
13.25	1.32	1.31			

Table SM.II. Selected values of the PIMC pair structures (no B-spline smoothing) for the QHS state points at $(\rho_N^* = 0.9, \lambda_B^* = 0.8)$ of the bcc-qIII (Ref. [67]) and cI16 (this work) solids. Distances r in Å, corresponding to the model $M = 28.0134$ amu, $\sigma = 3.5$ Å. Sample sizes $(N_S \times P)$ shown. Most g_2 values rounded off to two decimals.

Centroid - $g_{CM2}(r)$			Instantaneous - $g_{ET2}(r)$		
r/Å	bcc-qIII (432×24)	cI16 (1024×24)	r/Å	bcc-qIII (432×24)	cI16 (1024×24)
3.65	0	0	3.5	0	0
3.85	1.03	1.03	3.595	0.46	0.46
4.05	11.10	11.10	3.65	1.14	1.14
4.15	3.85	3.85	3.95	4.70	4.70
4.55	4×10^{-5}	5×10^{-6}	4.05	4.48	4.48
5.05	1.70	1.70	4.65	0.25	0.25
5.65	5×10^{-8}	5×10^{-7}	5.05	0.72	0.72
6.15	2.79	2.79	5.55	0.16	0.16
6.45	3×10^{-3}	2×10^{-3}	6.15	1.03	1.03
6.95	3.18	3.19	6.45	0.66	0.66
7.25	0.01	0.01	6.95	1.58	1.58
7.55	3.24	3.24	7.25	1.06	1.06
7.75	1.00	1.00	7.75	1.82	1.82
7.95	2.15	2.15	8.65	0.15	0.15
8.05	0.94	0.94	10.05	1.57	1.57
8.15	1.40	1.40	11.15	0.65	0.65
8.65	1×10^{-6}	2×10^{-6}	11.85	1.37	1.37
9.95	3.29	3.29	13.15	1.32	1.32
11.25	0.18	0.18	14.65	----	0.71
11.85	2.98	2.99	15.05	----	1.13

Table SM.III. Selected PIMC convergence features. Positions[a] r_{FP}^{*} and heights g in the close vicinities of the first maxima of the radial g_2 and equilateral $g_3^{EQ} = g_3(r^*, r^*r^*)$ correlations for the centroid (CM) and instantaneous (ET) cases. Number in parentheses stand for one-standard deviation in the last digit(s) written (e.g., 3.24(0) means that the error bars affect the third decimal place of the preceding figure; 3.27(1)≡ 3.27±0.01; 160.0(15)≡ 160.0±1.5).

λ_B^*	ρ_N^*	$N_S \times P$	r_{FP}^*, g_{CM2}	r_{FP}^*, g_{ET2}	r_{FP}^*, g_{CM3}^{EQ}	r_{FP}^*, g_{ET3}^{EQ}
FLUID PHASE (fluid-FCC coexistence line)						
0.8	0.533	864×12	1.27, 3.24(1)	1.27, 2.20(0)	1.27, 28.6(4)	1.27, 10.0(0)
		864×24	1.27, 3.27(1)	1.27, 2.20(0)	-----	-----
1.9832	0.348	864×30	1.5, 2.81(1)	1.47, 1.67(0)	1.5, 19.7(5)	1.47, 4.51(2)
		864×40	1.5, 2.82(1)	1.47, 1.67(0)	1.5, 19.9(5)	1.47, 4.53(3)
FCC PHASE (fluid-FCC coexistence line)						
0.8	0.573	864×12	1.3, 3.83(1)	1.3, 2.49(0)	1.3, 55.6(4)	1.3, 15.4(0)
		864×18	1.3, 3.84(1)	1.3, 2.49(0)	1.3, 56.3(5)	1.3, 15.4(0)
						FCC PHASE
0.2	0.925	864×12	1.1, 5.26(0)	1.13, 4.51(0)	1.1, 160.1(7)	1.13, 93.4(2)
		864×24	1.1, 5.27(1)	1.13, 4.51(0)	1.1, 160.0(15)	1.13, 93.1(3)
0.8	0.9	864×12	1.16, 13.2(0)	1.16, 4.78(0)	1.16, 2339(7)	1.16, 112.2(1)
		864×24	1.16, 14.4(0)	1.16, 4.81(0)	1.16, 3028(16)	1.16, 114.7(2)
		864×36	1.16, 14.6(0)	1.16, 4.80(0)	1.16, 3177(24)	1.16, 114.1(2)
						cI16 PHASE
0.2	0.925	1024×12	1.1, 5.60(1)	1.1, 4.52(1)	1.1, 146.8(20)	1.1, 79.5(10)
		1024×24	1.1, 5.60(1)	1.1, 4.51(1)	1.1, 148.0(31)	1.1, 79.9(16)
0.8	0.9	1024×12	1.16, 11.99(2)	1.13, 5.60(0)	1.16, 1469(11)	1.13, 158.0(2)
		1024×24	1.16, 11.11(2)	1.13, 4.70(0)	1.16, 1233(10)	1.13, 96.7(2)
		1024×36	1.16, 11.36(5)	1.13, 4.71(0)	1.16, 1334(14)	1.13, 97.8(3)

[a]Actual values of the three-digit rounded-off distances r_{FP}^*: 1.13→3.95/3.5; 1.16→4.05/3.5; 1.27→4.45/3.5; 1.47→5.15/3.5.

TABLE SM.IV. Perfect lattice cI16 tabulation of the fractional displacement parameters x vs.their maximal structure factor $S_2^C(\boldsymbol{k}_{max})$ values.

x	$S_2^C(\boldsymbol{k}_{max})$	x	$S_2^C(\boldsymbol{k}_{max})$	x	$S_2^C(\boldsymbol{k}_{max})$
0	1	0.031	0.732339	0.050	0.428381
0.002	0.998737	0.032	0.717115	0.051	0.412819
0.005	0.992130	0.033	0.701685	0.052	0.397430
0.008	0.979957	0.034	0.686069	0.053	0.382232
0.010	0.968830	0.035	0.670289	0.054	0.367240
0.012	0.955374	0.036	0.654367	0.055	0.352469
0.015	0.931009	0.037	0.638325	0.056	0.337931
0.018	0.901935	0.038	0.622184	0.057	0.323642
0.020	0.880132	0.039	0.605965	0.058	0.309612
0.021	0.868553	0.040	0.589691	0.059	0.295855
0.022	0.856545	0.041	0.573382	0.060	0.282381
0.023	0.844127	0.042	0.557059		
0.024	0.831318	0.043	0.540743		
0.025	0.818136	0.044	0.524453		
0.026	0.804600	0.045	0.508212		
0.027	0.790732	0.046	0.492037		
0.028	0.776551	0.047	0.475948		
0.029	0.762078	0.048	0.459963		
0.030	0.747333	0.049	0.444102		

Table SM.V. Salient equilateral features of the PIMC triplet correlation functions on the fluid side of the fluid-solid coexistence line. Discretizations selected at $\lambda_B^* = 0.8$ and $\lambda_B^* = 1.9832$: $P = 12$ and $P = 30$, respectively. Results in the close vicinities of the first maximum (FP), the first minimum (FV), and the second maximum (SP). $r^* = r/\sigma$. Numbers in parentheses stand for one-standard deviation affecting the last decimal(s) (e.g., 37.25(21) ≡ 37.25±0.21).

FLUID State Points				$g_3(r*, r*, r*) - \mathrm{CM3}$ (Centroid)			
λ_B^*	ρ_N^*	r_{FP}^*	$g_3(FP)$	r_{FV}^*	$g_3(FV)$	r_{SP}^*	$g_3(SP)$
0.2	0.789	1.1	62.81 (57)	1.5857	0.135 (16)	2.1571	2.405 (34)
0.4	0.672	1.1571	40.85 (40)	1.6714	0.136 (12)	2.2714	2.307 (36)
0.6	0.589	1.2429	32.12 (32)	1.7571	0.135 (13)	2.3857	2.247 (36)
0.8	0.533	1.2714	28.65 (37)	1.8143	0.119 (11)	2.4714	2.237 (35)
1.2543	0.442	1.3857	25.19 (42)	1.9286	0.105 (16)	2.6	2.255 (54)
1.9832	0.348	1.5	19.74 (50)	2.0714	0.102 (16)	2.7286	2.246 (59)
					$g_3(r*, r*, r*) - \mathrm{ET3}$		(Instantaneous)
0.2	0.789	1.1	35.75 (21)	1.5857	0.138 (5)	2.1571	2.327 (18)
0.4	0.672	1.1857	19.27 (9)	1.6714	0.151 (4)	2.2714	2.142 (14)
0.6	0.589	1.2429	12.93 (6)	1.7571	0.165 (4)	2.3571	2.029 (12)
0.8	0.533	1.3	10.03 (4)	1.8143	0.174 (4)	2.4143	1.966 (9)
1.2543	0.442	1.3857	6.82 (3)	1.9286	0.223 (3)	2.5571	1.826 (13)
1.9832	0.348	1.4714	4.51 (2)	2.1286	0.338 (4)	2.7857	1.594 (11)

Table SM.VI. Salient equilateral features of the PIMC triplet correlation functions on the FCC solid side of the fluid-solid coexistence line. Discretization selected at $\lambda_B^* = 0.8$: $P = 12$. Results in the close vicinities of the first maximum (FP), the first minimum (FV), and the second maximum (SP). $r^* = r/\sigma$. Numbers in parentheses stand for one-standard deviation affecting the last decimal(s) (e.g., 55.64(14) ≡ 55.64±0.14).

FCC State Points				$g_3(r*, r*, r*)$ − CM3 (Centroid)			
λ_B^*	ρ_N^*	r_{FP}^*	$g_3(FP)$	r_{FV}^*	$g_3(FV)$	r_{SP}^*	$g_3(SP)$
0.2	0.863	1.1	83.63 (51)	1.5857	8×10^{-5}	2.0143	5.924 (63)
0.4	0.731	1.1857	66.86 (43)	1.6714	2×10^{-5}	2.1286	5.386 (56)
0.6	0.635	1.2429	55.60 (38)	1.7571	0.0001	2.2429	4.952 (64)
0.8	0.573	1.3	55.56 (40)	1.7857	7×10^{-5}	2.3286	5.261 (63)
1.2543	0.465	1.4143	48.32 (66)	1.9286	0.0001	2.5	4.899 (106)
1.9832	0.360	1.5286	39.89 (83)	2.1	0.0004	2.7	4.014 (130)
						$g_3(r*, r*, r*)$ − ET3	(Instantaneous)
0.2	0.863	1.1286	55.64 (14)	1.5857	7×10^{-5}	2.0143	5.205 (34)
0.4	0.731	1.1857	30.78 (9)	1.6714	0.0005 (1)	2.1286	3.805 (20)
0.6	0.635	1.2714	19.96 (7)	1.7571	0.0024 (2)	2.2429	2.946 (14)
0.8	0.573	1.3	15.42 (5)	1.8143	0.0055 (4)	2.3286	2.574 (10)
1.2543	0.465	1.3857	9.18 (3)	1.9286	0.0374 (9)	2.4714	2.006 (5)
1.9832	0.360	1.5	5.44 (2)	2.1	0.159 (3)	2.7	1.630 (5)

Table SM.VII. PIMC isosceles results for $g_3(r^*, s^*, s^*)$-cI16, in the region of moderately high densities at selected r^* slices. Values r^* correspond to salient equilateral features in the close vicinities of the first peak (FP), center of first valley (FV), and third peak (TP). Most values rounded off to two decimal places. $r^* = r/\sigma,\ s^* = s/\sigma$.

cI16 ($\lambda_B^* = 0.2; \rho_N^* = 0.925$) PIMC-P=12						
	Centroid–$g_{CM3}(r^*, s^*, s^*)$			Instantaneous–$g_{ET3}(r^*, s^*, s^*)$		
s^*	$r_{FP}^* = 1.1$	$r_{FV}^* = 1.4714$	$r_{TP}^* = 1.9$	$r_{FP}^* = 1.1$	$r_{FV}^* = 1.4714$	$r_{TP}^* = 1.9$
1	0	0	0	0	0	0
1.0143	0	0	0	0.65	0.05	0.02
1.0429	1.69	0.24	0.26	16.73	2.16	1.95
1.0714	83.75	13.30	23.42	55.04	9.45	16.89
1.1	146.80	25.58	54.65	79.46	15.90	33.46
1.1286	106.83	19.18	32.49	72.65	15.39	26.49
1.1571	63.91	11.09	11.90	50.48	10.48	12.51
1.2143	17.15	2.27	1.91	15.59	2.55	2.39
1.4143	0.03	1×10^{-4}	2.97	0.04	3×10^{-4}	2.48
1.4714	3×10^{-3}	0	1.87	6×10^{-3}	4×10^{-5}	1.61
1.5571	3×10^{-3}	4×10^{-4}	0.11	6×10^{-3}	9×10^{-4}	0.14
1.7571	0.04	7×10^{-3}	2.62	0.08	0.01	2.35
1.8143	0.44	7×10^{-3}	2.18	0.59	0.01	2.24
1.9	5.03	0.02	2.91	4.02	0.04	2.61
1.9286	7.02	0.05	2.76	5.32	0.08	2.45
1.9571	7.89	0.13	2.22	6.00	0.16	2.09
1.9857	7.52	0.26	1.68	5.95	0.31	1.73
2.0143	6.52	0.49	1.44	5.44	0.52	1.57
2.1857	15.87	1.82	3.58	11.86	1.66	3.38
2.3857	1.06	0.70	0.49	1.03	0.64	0.48

Table SM.VIII. PIMC isosceles results $g_3(r^*, s^*, s^*)$-FCC, in the region of moderately high densities at selected r^* slices. Values r^* correspond to salient equilateral features in the close vicinities of the first peak (FP), center of first valley (FV), and second peak (SP). Most values rounded off to two decimal places. $r^* = r/\sigma$, $s^* = s/\sigma$.

FCC ($\lambda_B^* = 0.2; \rho_N^* = 0.925$) PIMC-P=12						
	Centroid–$g_{CM3}(r^*, s^*, s^*)$			Instantaneous–$g_{ET3}(r^*, s^*, s^*)$		
s^*	$r_{FP}^* = 1.1$	$r_{FV}^* = 1.5571$	$r_{SP}^* = 1.9857$	$r_{FP}^* = 1.1286$	$r_{FV}^* = 1.5571$	$r_{SP}^* = 1.9857$
1	0	0	0	0	0	0
1.0143	0	0	0	0.49	0.07	7×10^{-5}
1.0429	1.31	0.24	8×10^{-3}	14.15	3.06	0.08
1.0714	76.89	16.92	4.01	53.06	14.00	4.12
1.1	160.07	36.76	38.49	88.42	23.62	28.13
1.1286	134.68	28.42	72.28	93.38	22.30	55.45
1.1571	90.37	15.67	56.22	74.23	14.46	47.52
1.2143	27.35	2.48	5.86	27.58	2.89	6.70
1.4143	5×10^{-3}	5×10^{-6}	5×10^{-3}	0.01	5×10^{-5}	0.01
1.4714	1×10^{-4}	0	1×10^{-3}	5×10^{-4}	2×10^{-6}	3×10^{-3}
1.5571	6×10^{-6}	0	2×10^{-5}	3×10^{-5}	0	1×10^{-4}
1.6429	6×10^{-5}	0	2×10^{-4}	2×10^{-4}	2×10^{-6}	7×10^{-4}
1.7571	0.02	4×10^{-3}	0.02	0.04	0.01	0.05
1.9286	22.32	8.47	8.33	16.59	7.41	7.06
1.9857	30.86	11.22	13.53	23.05	9.47	10.80
2.0429	19.13	6.23	8.22	15.94	5.72	7.09
2.1	6.14	1.69	2.23	6.10	1.81	2.29
2.1571	1.18	0.24	0.36	1.45	0.31	0.47
2.2143	0.18	0.02	0.07	0.27	0.03	0.11
2.3857	0.73	0.10	0.19	0.76	0.13	0.27

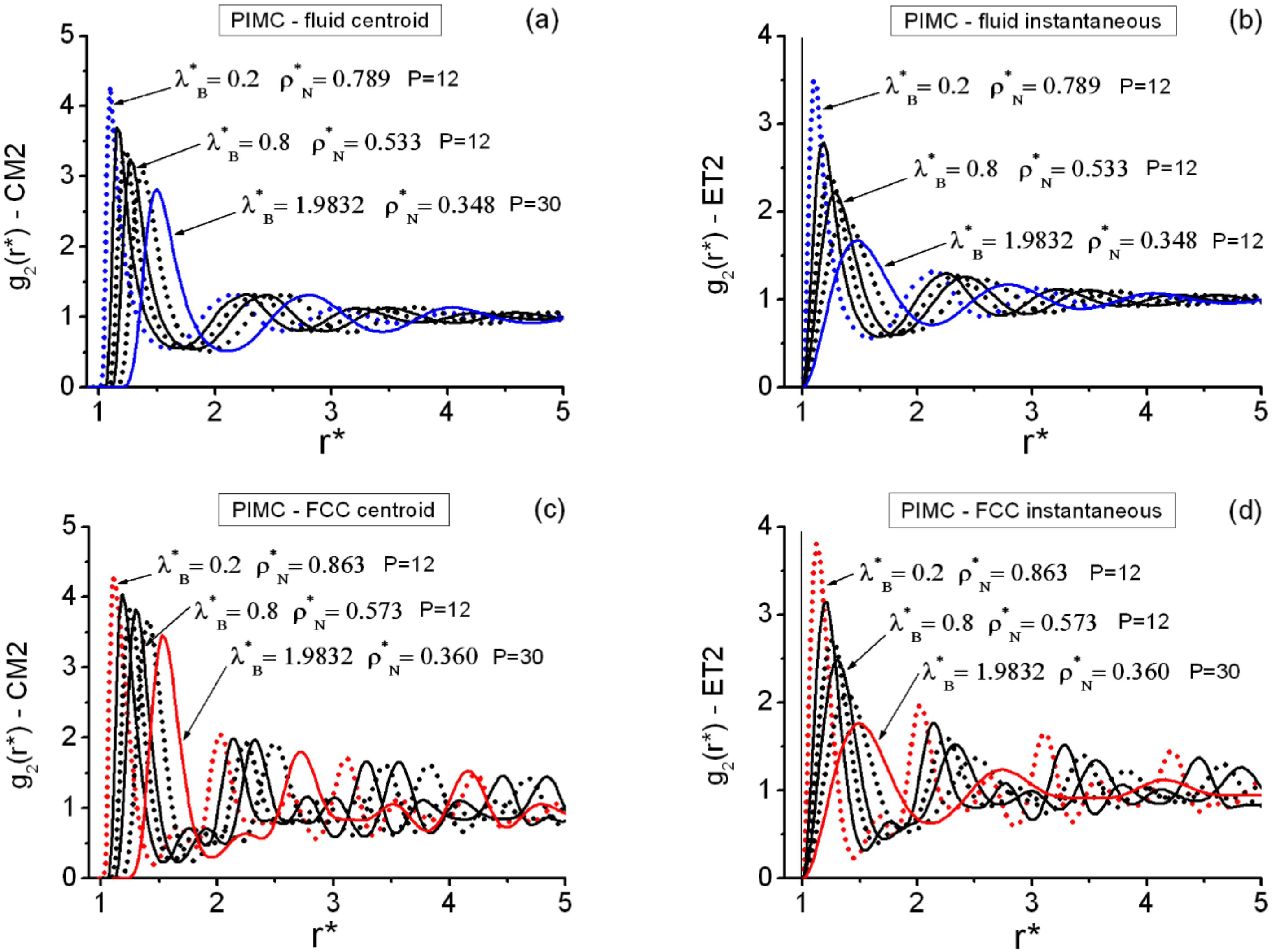

Figure SM 1. PIMC pair radial functions along the fluid-FCC coexistence line of the quantum hard-sphere system. See the main text for the (ρ_N^*, λ_B^*) values of the arrangement of the six state points on each side of the coexistence line. The vertical line in (b) and (d) at $r^* = 1$ marks the position of the hard core.

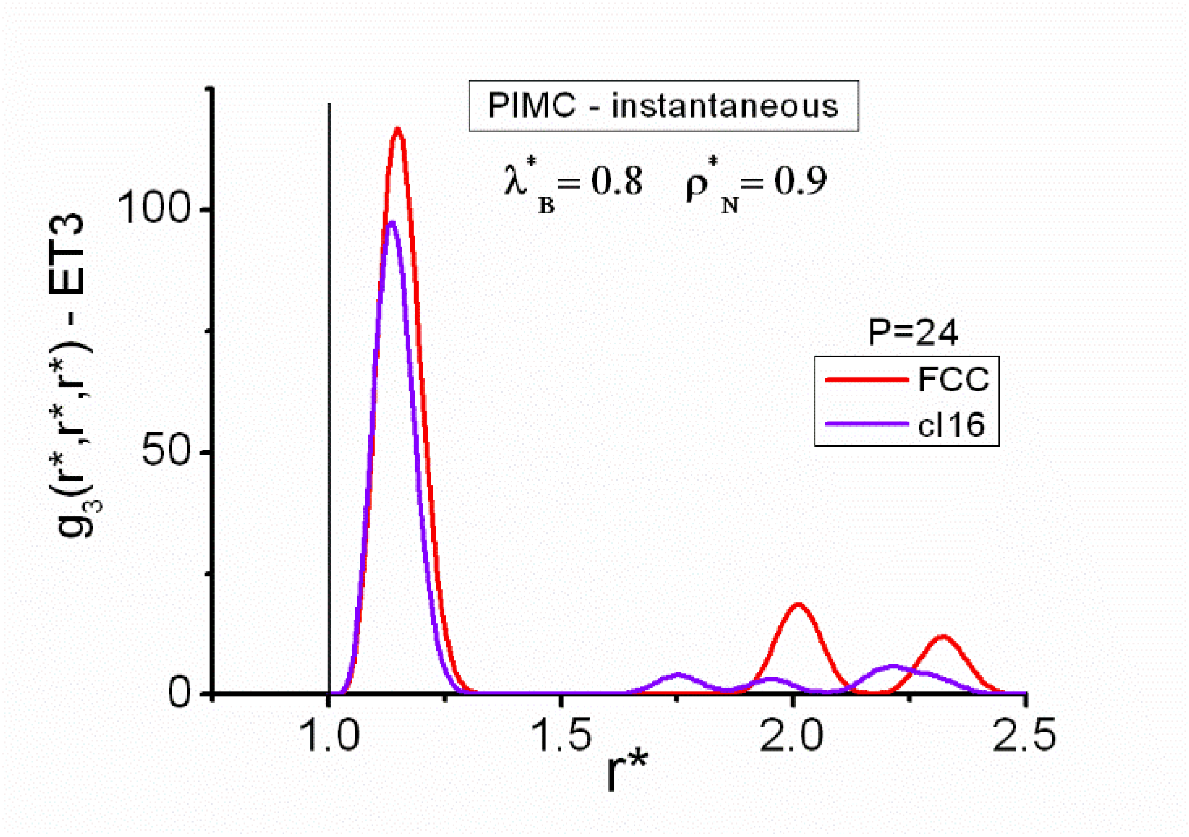

Figure SM 2. PIMC instantaneous equilateral structures of FCC and cI16 in the region of moderately high densities. The vertical line at $r^* = 1$ marks the position of the hard core.
